# Expression dynamics of *Mage* family genes during self-renewal and differentiation of mouse pluripotent stem and teratocarcinoma cells

**DOI:** 10.18632/oncotarget.26933

**Published:** 2019-05-14

**Authors:** Olga Gordeeva, Andrey Gordeev, Sergey Khaydukov

**Affiliations:** ^1^Kol'tsov Institute of Developmental Biology, Russian Academy of Sciences, Moscow, Russia; ^2^National Institutes of Health's National Library of Medicine, Bethesda, MD, USA; ^3^Medical Science and Computing Bethesda, Bethesda, MD, USA; ^4^M.M. Shemyakin and Yu.A. Ovchinnikov Institute of Bioorganic Chemistry, Russian Academy of Sciences, Moscow, Russia

**Keywords:** melanoma antigens, cancer-testis antigens, teratocarcinoma, pluripotent stem cells, differentiation

## Abstract

The biological roles of cancer-testis antigens of the Melanoma antigen (Mage) family in mammalian development, stem cell differentiation and carcinogenesis are largely unknown. In order to understand the involvement of the *Mage* family genes in maintenance of normal and cancer stem cells, the expression patterns of *Mage-a, Mage-b, Mage-d, Mage-e, Mage-h* and *Mage-l* gene subfamilies were analyzed during the self-renewal and differentiation of mouse pluripotent stem and teratocarcinoma cells. Clustering analysis based on the gene expression profiles of undifferentiated and differentiating cell populations revealed strong correlations between *Mage* expression patterns and differentiation and malignant states. Gene co-expression analysis disclosed the potential contributions of *Mage* family members in self-renewal and differentiation of pluripotent stem and teratocarcinoma cells. Two gene clusters including *Mage-a4* and *Mage-a8, Mageb1, Mage-d1, Mage-d2, Mage-e1, Mage-l2* were identified as functional antagonists with opposing roles in the regulation of proliferation and differentiation of mouse pluripotent stem and teratocarcinoma cells. The identified aberrant expression patterns of *Mage-a2, Mage-a6, Mage-b4, Mageb-16* and *Mage-h1* in teratocarcinoma cells can be considered as specific teratocarcinoma biomarkers promoted the malignant phenotype. Our study first provides a model for the involvement of *Mage* family members in regulatory networks during the self-renewal and early differentiation of normal and cancerous stem cells for further research of the predicted functional modules and the development of new cancer treatment strategies.

## INTRODUCTION

Cancer-testis antigens (CTAs) are among the most enigmatic genes in animal and human genomes because their expression patterns are highly specific, but their cell functions remain unknown. CTAs are abundantly expressed in different types of cancer cells and involved in the regulation of proliferation [1–6], apoptosis [5, 7–9], the epithelia-mesenchymal transition [10, 11], and germ [12–16] and somatic cell [17–23] differentiation. Several CTA families’ members can induce spontaneous humoral and cytotoxic T-cell-mediated immune responses in cancer patients. Their immunotheraputic potential was studied in numerous clinical trials with CTA-based cancer vaccines [24–26].

The CTAs’ roles in normal and pathological cell processes are largely unclear [27–31]. In addition to cancer cells and testes [6, 16, 18, 28, 32], CTA expression was identified during the development of extra-embryonic structures and embryonic germ and somatic cells [17, 19–23, 33], as well as during pluripotent and multipotent stem cell differentiation [14, 28, 34, 35]. Previous studies revealed different CTA expression patterns in normal and cancer stem cells [34, 36–40]. Therefore, we hypothesized that CTA expression may be a part of the developmental programs of both the germ and somatic lineages. Changes in the characteristic CTA profiles (aberrant expression) of germ and somatic cells may be associated with abnormal differentiation and cancer transformation.

The CTAs of the Melanoma antigen (*Mage*) family identified in the human and mouse genomes belong to two classes, according to their expression patterns: *Mage-a* and *Mage-b* genes, which are expressed predominantly in spermatogenic and cancer cells; and the ubiquitously expressed *Mage-d, Mage-e, Mage-h1,* and *Mage-l2* genes [20, 28, 29, 41–43]. Like other CTAs, Mage proteins are considered intrinsically disordered proteins that can transition to an ordered 3D-structure and can interact with nucleic acids or target proteins involved in the regulation of different cell processes [44]. Mage family proteins are widely involved in cancer progression and may be a driver of tumorigenesis [45]. Notwithstanding significant clinical interest in Mage antigens, the expression patterns and putative functional roles of the Mage family in mammalian development, cell differentiation and cancer transformation are poorly understood. Because of the high homology and co-expression of the *Mage* family genes, traditional studies using the gain- or loss-of-function mutations and gene knockdown approaches for individual members of the Mage family were insufficient for the disclosure of the functional role of the Mage family in normal and pathological cell processes. Therefore, gene co-expression analysis of the *Mage* family members and key regulator of pluripotency, self-renewal and differentiation may shed light on the unknown biological roles and functional importance of these genes in normal and cancer cells.

The present study is the first systematic co-expression analysis of both classes of *Mage* family genes in mouse embryonic stem (ESCs), embryonic germ (EGCs) and teratocarcinoma (ECCs) cells and early embryos in order to understand the possible involvement of *Mage* family genes in the regulation of stem cell self-renewal and differentiation and carcinogenesis. Possible functionally-related Mage gene modules were identified via clustering and analyzing the correlations between the expression patterns of the *Mage* genes and regulators of proliferation and differentiation in pluripotent stem and teratocarcinoma cells.

## RESULTS

### *Mage* family expression profiles differ during self-renewal of pluripotent stem and malignant teratocarcinoma cells

**Figure 1:**
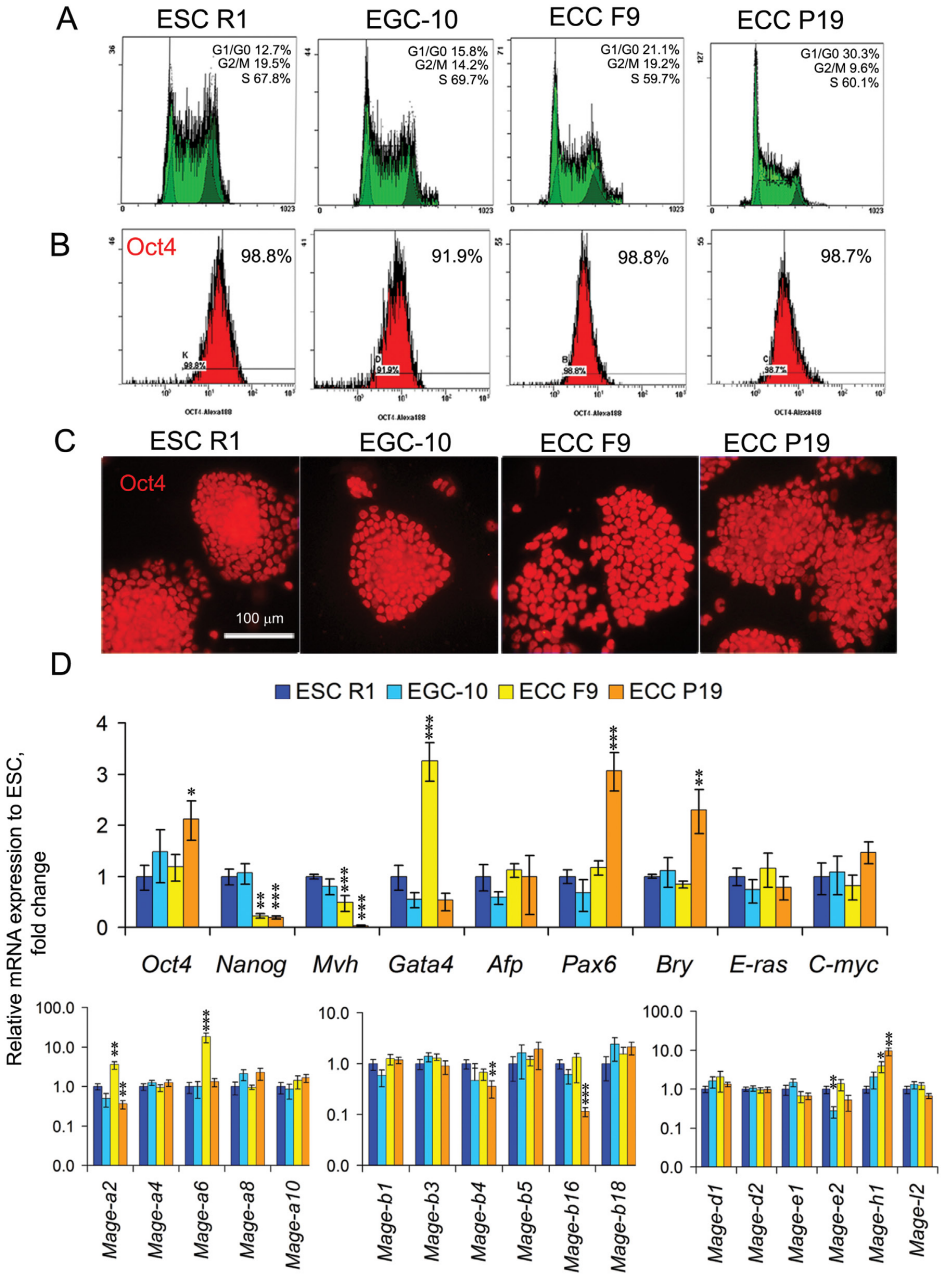
Characteristics and marker and Mage family gene expression patterns in undifferentiated ESCs, EGCs and ECCs. (**A**) Flow cytometric analysis of the distributions of the cell cycle stages and (**B**) number of Oct4-expressing cells in the populations of undifferentiated ESCs, EGCs and ECCs. (**C**) Staining of undifferentiated ESCs, EGCs and ECCs with antibodies against Oct4. Scale bar = 100 μm. (**D**) Quantitative PCR analysis of the expression profiles of markers of proliferative activity (*C-myc, E-ras*), pluripotency (*Oct4, Nanog*), embryonic lineages (*Mvh, Gata4, Afp, Pax6, Bry*) and *Mage* family genes in undifferentiated ESCs, EGCs and ECCs. The gene expression levels (fold change) in EGCs, ECCs F9 and ECCs P19 were evaluated relative to the gene expression levels in ESCs R1. The data are represented as the means ± s.d., ^*^p < 0.05, ^**^p < 0.01, ^***^p < 0.01, ANOVA.

In the undifferentiated ESCs R1, EGCs EGC-10, ECCs F9 and ECCs P19 there are similar cell cycle distributions, with most cells in the S-phase of the cell cycle (60–70%) and low number of cells in the G1-phase of the cell cycle (10–30%) (Figure 1A). Undifferentiated cells express *E-ras* and *C-myc* at similar levels and have similar expression patterns for the pluripotent stem cell marker *Oct4* (Figure 1B–1D, Supplementary Table 1). However, the expression levels of the pluripotency markers *Oct4* and *Nanog* and lineage-specific gene markers *Mvh, Gata4, Pax6* and *Bry* significantly differ between pluripotent stem and teratocarcinoma cells (Figure 1D, Supplementary Table 1).

The undifferentiated ESCs, EGCs and ECCs exhibit differential expression patterns for 6 of the 17 *Mage* genes (35%) and similar expression patterns for 11 *Mage* genes (65%) studied (Figure 1D, Supplementary Table 1). Only *Mage-e2* is expressed at different levels in pluripotent EGCs and ESCs (*p* < 0.01), whereas the expression patterns of five *Mage* genes differs between pluripotent and teratocarcinoma cells. Both ECC lines express significantly higher level of *Mage-h1* (*p* < 0.01) than pluripotent stem cells. *Mage-a2* and *Mage-a6* are expressed at significantly higher levels in ECCs F9 and *Mage-a2, Mage-b4* and *Mage-b16* are expressed at significantly lower levels in ECCs P19 than in pluripotent stem cells. The Mage family proteins are expressed in the majority of undifferentiated ESCs, EGCs and ECCs regardless of the cell cycle phase, and no substantial differences in the intensity or localization of Mage immunostaining signals in cells in different phases of the cell cycle were observed. (Supplementary Figure 1). Thus, despite significant similarity of the cell characteristics and expression level of proliferation regulators *C-myc* and *E-ras*, aberrant expression patterns for 11 of 26 (42%) Mage and marker genes were identified when comparing undifferentiated self-renewing pluripotent stem and teratocarcinoma cells.

### Expression patterns of *Mage* family genes in ESCs, EGCs and ECCs dynamically change during the RA-stimulated differentiation

**Figure 2:**
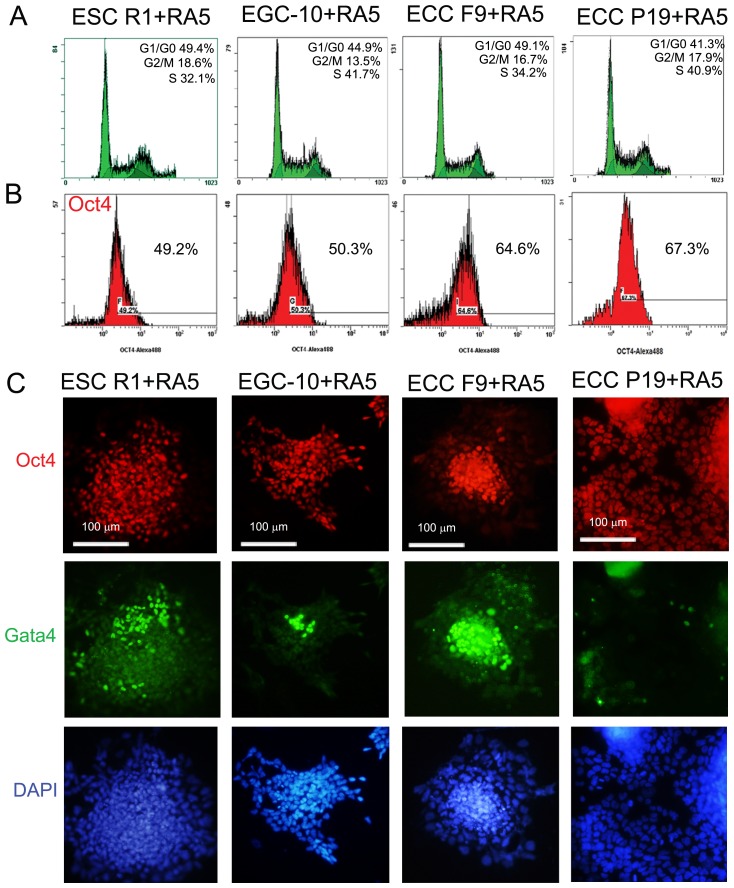
ESC, EGC and ECC population characteristics during RA-induced differentiation. (**A**) Flow cytometric analysis of the cell cycle distributions of differentiating ESCs, EGCs and ECCs after 5-day RA exposure. (**B**) Flow cytometric analysis of number of Oct4-expressing cells in the populations of differentiating ESCs, EGCs and ECCs after 5-day RA exposure. (**C**) Triple staining of differentiating ESCs, EGCs and ECCs with antibodies against Oct4 and Gata4. Nuclei were counterstained by DAPI. Scale bar = 100 μm.

**Figure 3:**
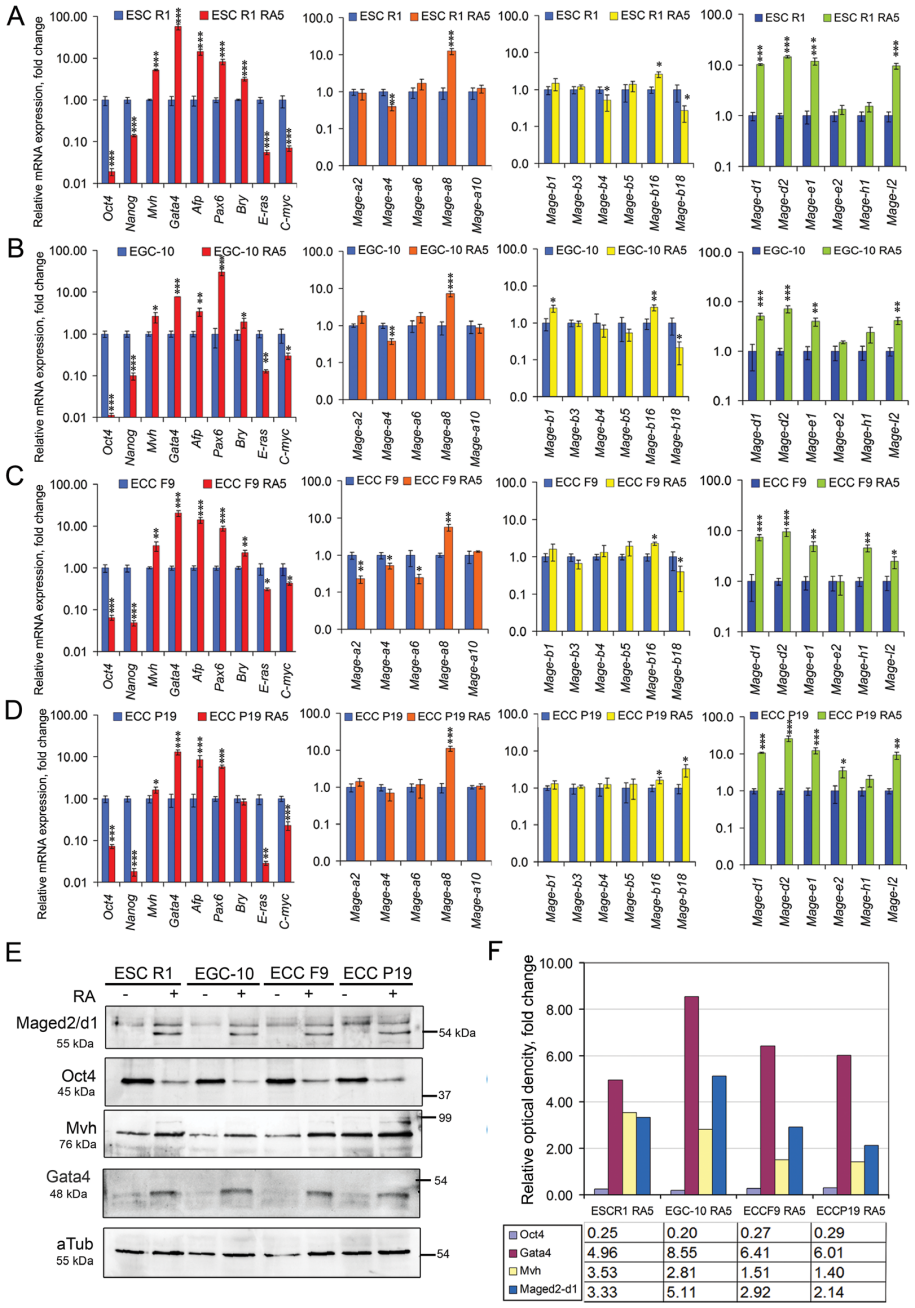
Dynamics of *Mage* and marker gene expressions during RA-induced differentiation of ESCs, EGCs and ECCs. The gene expression levels of each gene in differentiating cells were evaluated relatively to the gene expression levels in undifferentiated ESCs R1 (**A**), EGCs EGC-10 (**B**), ECCs F9 (**C**) and ECCs P19 (**D**), respectively. The data are represented as the means ± s.d. ^*^p < 0.05, ^**^p < 0.01, ^***^p < 0.01, ANOVA. (**E**) Expression of the Mage-d1/d2 proteins, Oct4, Gata4 and Mvh in undifferentiated and RA-stimulated ESCs R1, EGCs-10, ECCs F9 and ECCs P19 as determined by western blotting; a-tubulin (aTub) was used as the loading control (uncropped blots are represented in Supplementary Figure 3). The representative of experiments repeated at least three times is shown. (**F**) Densitometric evaluation of bands intensity from (**D**). Figure 6F shows relative expression of Mage-d1-d2, Oct4, Gata4 and Mvh in RA-stimulated ESCs R1, EGCs-10, ECCs F9 and ECCs P19 calculated in comparison to respective undifferentiated cells. The relative protein expression levels were normalized to a-tubulin (aTub) of corresponding samples. Summary relative intensity of both bands for Mage-d1-d2 proteins were evaluated for RA-stimulated cells.

The differentiation potential of the pluripotent stem cells, ESCs R1 and EGCs-10, and teratocarcinoma cells, ECCs F9 and ECCs P19, was significantly different during spontaneous *in vitro* differentiation and after transplantation into Nude mice [46–48]. However, during RA-induced differentiation, the ESCs, EGCs and ECCs exhibited similar, nearly two-fold decreases in the number of cells in the S-phase of the cell cycle (30–40%) and the number of Oct4-expressing cells (50–60%) (Figure 2A–2C). In all cell lines, RA stimulates a significant down-regulation of *E-ras*, *C-myc*, *Oct4* and *Nanog* expression and up-regulation of the expression of the germ and somatic lineage markers *Mvh,* *Pax6, Afp*, *Bry* and *Gata4* (Figures 2C and 3, Supplementary Tables 1 and 2).

The dynamics of *Mage* gene expression patterns during the RA-induced differentiation of ESCs, EGCs, and ECCs have similar trends for all cell lines. The expression of 4 of 6 ubiquitously expressed genes, *Mage-d1*, *Mage-d2*, *Mage-e1* and *Mage-l2,* increases dramatically in all cell lines, *Mage-e2* and *Mage-h1* expression increases in ECCs P19 and F9, respectively, while the expression of only 6 of 11 genes of the *Mage-a* and *Mage-b* subfamilies (*Mage-a4, Mage-a8*, *Mageb-1, Mage-b4, Mage-b16,* and *Mage-b18)* changes significantly in differentiating cells (Figure 3A–3D, Supplementary Tables 1 and 2). Moreover, significant differences in the expression of *Mage-a2*, *Mage-a6* (ECCs F9) and *Mage-b18* (ECCs P19) were found between cell lines during RA-induced differentiation (Figure 3).

The expression of Mage proteins was detected in undifferentiated and differentiating cells using the polyclonal rabbit anti-Mage antibodies obtained to an epitope from the C-terminal region of human MAGE-A1 antigen (sc-10749), which can cross-react with a wide range of mouse Mage family antigens due to their high homology (Supplementary Figure 1). However, these antibodies were not suitable for a quantitative evaluation of the dynamics of Mage protein expression during RA-induced differentiation. Therefore, antibodies against Mage-d1/d2 antigens (NBP2-24694), which mRNA has changed most significantly during differentiation, were used for semi-quantitative western blot analysis. A western blot detected bands with molecular weights in the predicted area (55 kDa), which correspond to the Mage-d2 isoforms X1 and X2 (XP_006529062.1, 616 aa and XP_011246184.1, 594 aa, respectively) and the Mage-d1 isoform CRA_a (EDL29758.1; 592 aa) (Figure 3E). Moreover, two bands with molecular weights between 85–100 kDa, which may correspond to the Mage-d1 isoform CRA_b (EDL29757.1, NP_062765.1; 775aa, 85 kDa predicted) as well as Mage-e1 (918aa, 102 kDa predicted) were detected in all cells (Supplementary Figures 2 and 3). Semi-quantitative densitometry evaluation of two bands in the predicted 55 kDa area showed 2–5 fold increase of Mage-d1/-d2 protein expression in differentiating cells of all cell lines (Figure 3E, 3F). Similarly, the relative optical density of bands in area of 85–100 kDa was 2–3 fold higher for all differentiating cells (Supplementary Figure 2). In addition, up-regulation of Gata4 and Mvh protein expression and down-regulation of Oct4 expression were found in differentiating cells (Figure 3E, 3F). Thus, the Mage-d1/d2 protein expression patterns are consistent with the mRNA expression pattern data and correlate with the expression patterns of Oct4, Gata4 and Mvh.

### *Mage* family gene expression patterns in E7.5 mouse embryos and differentiating ESCs show significant similarities

**Figure 4:**
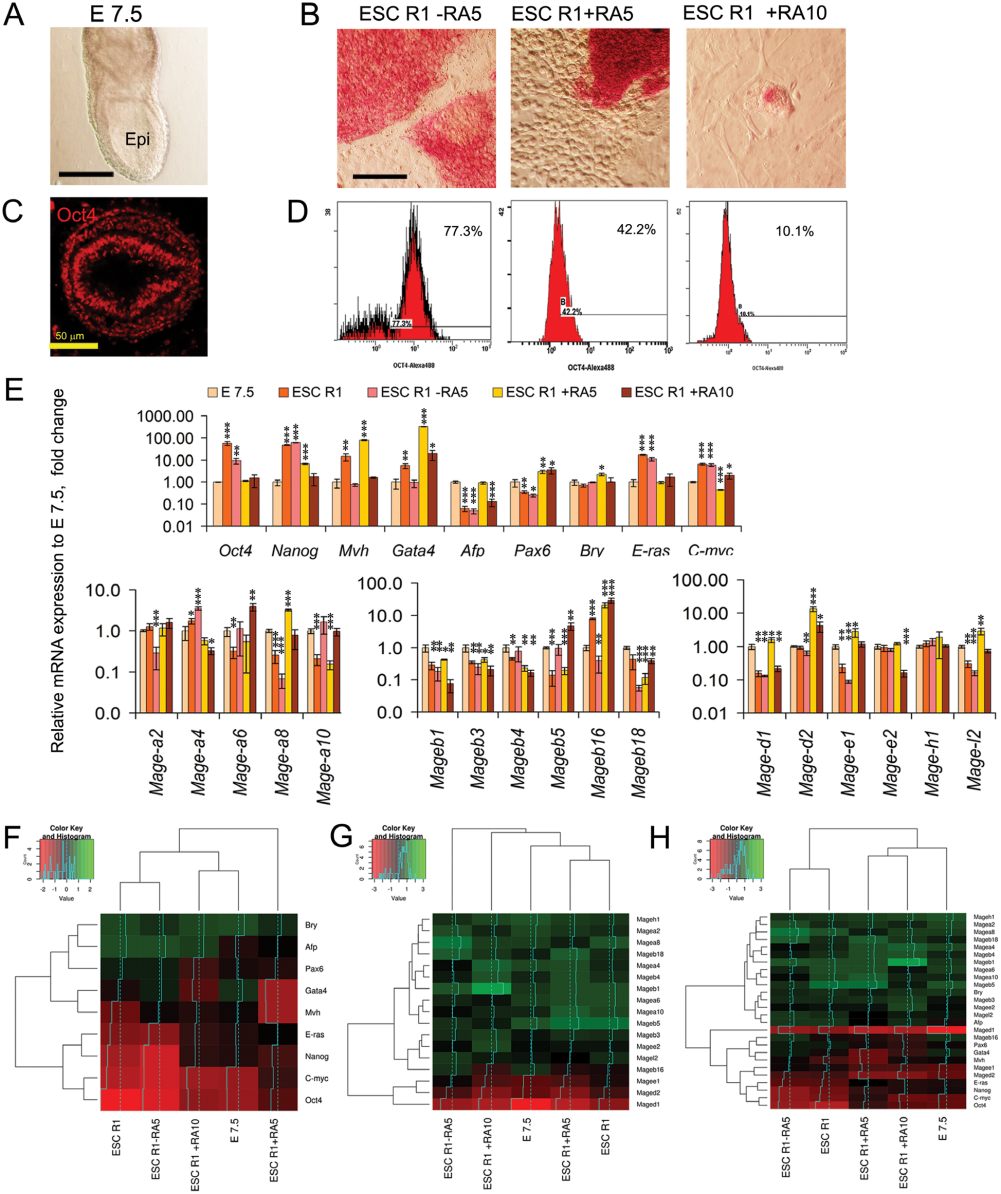
Comparative analysis of *Mage* family expression patterns in E7.5 mouse embryos and differentiating ESCs. (**A**) The epiblast of mouse embryos at the E7.5 stage. Scale bar = 100 μm. (**B**) ALP activity in the spontaneously and RA-induced (for 5 and 10 days) differentiating ESCs. Scale bar = 100 μm. (**C**) Immunofluorescent analysis of Oct4-expressing cells in the epiblast of E7.5 mouse embryos (cross-section). Scale bar = 50 μm. (**D**) Flow cytometric analysis of Oct4-expressing cells in the spontaneously and RA-induced (for 5 and 10 days) differentiating ESCs. (**E**) The expression patterns of regulators of proliferation, pluripotency and embryonic lineages and *Mage* genes in E7.5 embryos and spontaneously and RA-induced (for 5 and 10 days) differentiating ESCs. The gene expression levels in differentiating ESCs R1 were evaluated relative to the gene expression levels in embryos at the E7.5 stage. The data are represented as the means ± s.d., ^*^p < 0.05, ^**^p < 0.01, ^***^p < 0.01, ANOVA. (**F–G**). Heatmaps and hierarchical clustering dendrograms based on the data (the averaged ΔCt values) on mutual gene expression profiles (**H**), gene-markers (**F**) and *Mage* family gene expression profiles (**G**) demonstrate the similarity/dissimilarity between the gene expression patterns of the E7.5 embryos and differentiating ESC populations.

The consistency between the *Mage* expression patterns *in vivo* and *in vitro* was studied in mouse embryos at the E7.5 mid-gastrula stage and in undifferentiated and differentiating ESCs. The E7.5 embryos as well as various differentiating ESC populations contained undifferentiated cells and the early precursors of embryonic and extraembryonic cells (Figure 4A–4D). The heterogeneity of differentiating ESC populations was substantially different during spontaneous and RA-induced differentiation (Figure 4B, 4D). Among differentiating ESC populations, the largest number of Oct4- and ALP-positive cells was detected in spontaneously differentiating ESC R1 - RA5 (77%) cells and the lowest number (10.1%) was detected after RA exposure for 10 days in ESC R1+RA10 (Figure 4D). Quantitative analysis of the gene expression patterns and hierarchical clustering of the gene expression data sets revealed the greatest similarity gene expression patterns of E7.5 embryos with ESCs R1+RA10 for the markers (Figure 4E–4H; Supplementary Table 3) and with ESCs R1+RA10 and ESCs R1+RA5 for the *Mage* gene expression patterns (Figure 4E, 4G, 4H; Supplementary Table 3). The data on the expression patterns of 17 *Mage* genes in the E7.5 mouse embryos were annotated to the Gene Expression Database of Mouse Genome Informatics (http://www.informatics.jax.org/reference/J:230330).

### *Mage* gene expression patterns of ESCs, EGCs and ECCs and E7.5 mouse embryos correlate with differentiation and malignant states

**Figure 5:**
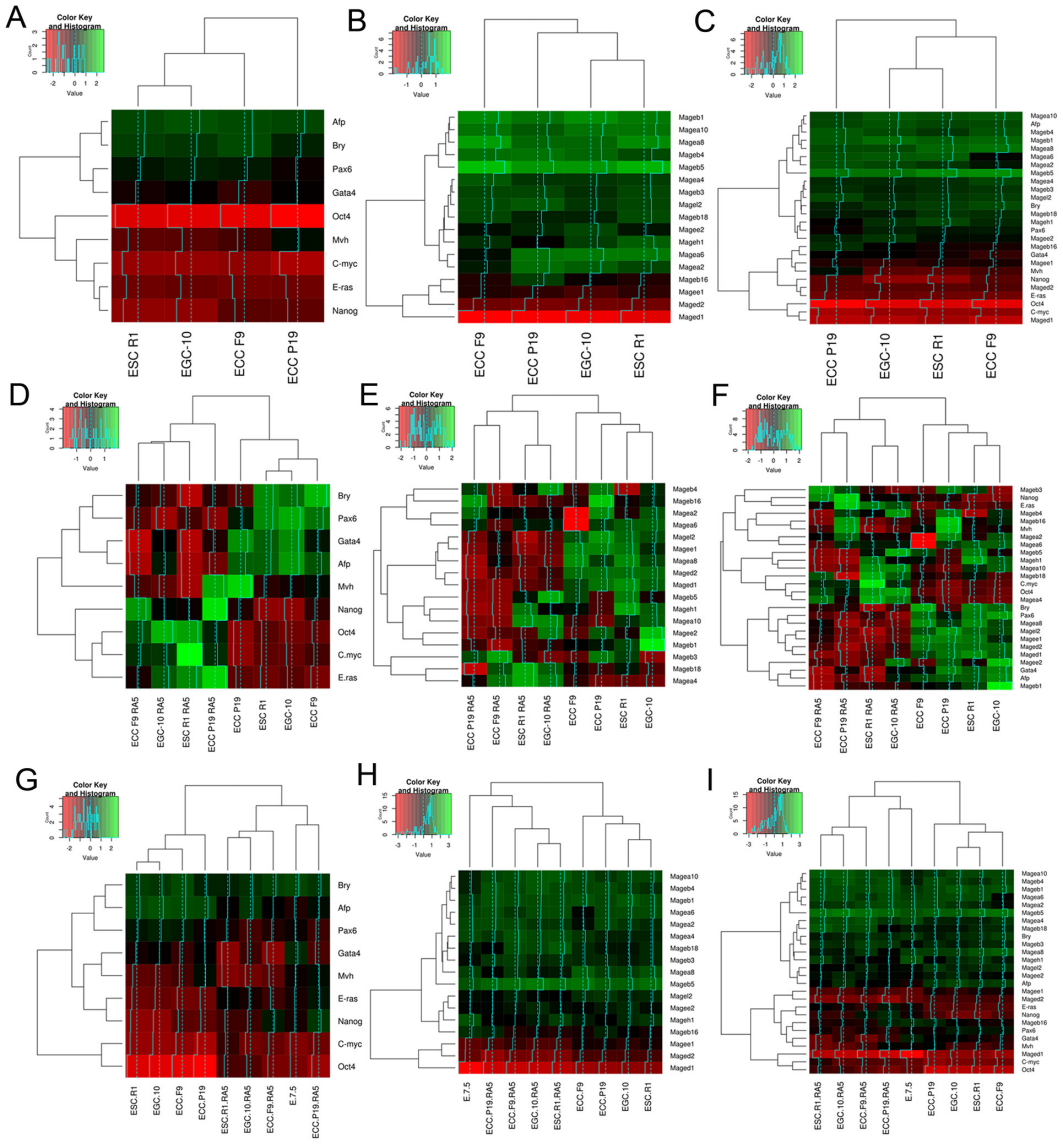
Heatmaps and hierarchical clustering dendrograms for gene expression patterns of ESC, EGC, ECC and E7.5 embryo. The results of expression pattern analyses for gene-markers (**A, D, G**), Mage family genes (**B, E, H**) and mutual gene expression patterns (**C, F, I**) in undifferentiated and differentiating ESCs, EGCs and ECCs and E7.5 embryos. (**A**–**C**) Dendrograms show dissimilarities of the *Mage* family profiles in normal pluripotent stem (ESCs R1 and EGCs-10) and teratocarcinoma cells (ECCs F9 and P19). (**D**–**F**) Dendrograms show strong consistency of the *Mage* expression patterns and differentiation states of ESCs, EGCs and ECCs. (**G**–**I**) The similarity of the *Mage* family and marker expression profiles of differentiating ESCs and E 7.5 embryos displays the consistency of the gene expression patterns during *in vitro* and *in vivo* differentiation of normal and malignant pluripotent stem cells.

Gene expression analysis has demonstrated significant changes in *Mage* family expression patterns during differentiation and early development. To test the possibility of classifying cell populations based on their *Mage* gene expression patterns, the hierarchical clustering based on ΔCt values was applied for three gene expression data sets: marker genes, *Mage* genes, and markers + *Mage* genes. Hierarchical clustering of the gene expression profiles of undifferentiated pluripotent stem and teratocarcinoma cells revealed a similarity of the profiles of normal ESCs R1 and EGCs-10, whereas ECCs F9 and ECCs P19 were less similar to pluripotent cells as well as to each other. The cluster dendrograms were similar for both the expression profiles of markers and *Mage* family genes (Figure 5A–5C). Thus, the Mage expression profile, to the same extent as the marker expression profile, reflects aberrant gene expression in teratocarcinoma cells compared to normal stem cells. Moreover, hierarchical clustering of the gene expression profiles of undifferentiated and differentiating ESCs, EGCs, and ECCs, as well as the E7.5 embryos, revealed a clear segregation of the studied cellular populations into two groups according to their differentiation states (Figure 5D–5I).

### The involvement of *Mage* family members in the self-renewal and differentiation of pluripotent stem and teratocarcinoma cells

**Figure 6:**
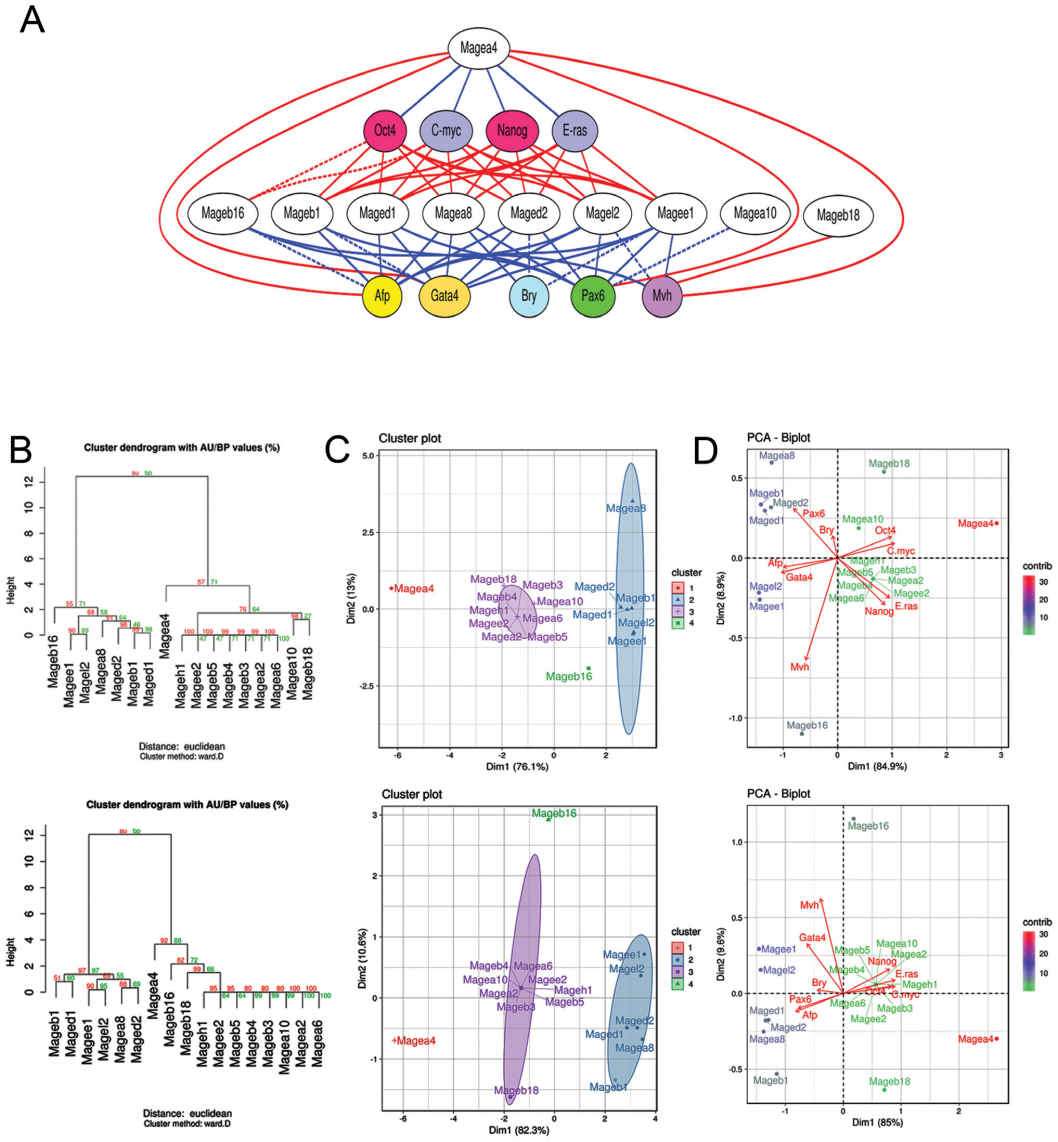
*Mage* family contributions in the self-renewal and differentiation of ESCs, EGCs, ECCs and E7.5 embryos. (**A**) Visualization of gene co-expression analysis based on significant strong and moderate correlations between the expression patterns of *Mage* family genes and pluripotency, proliferation and lineage markers using the Graphviz software. Blue and red lines indicate positive and negative correlations, relatively. Solid lines indicate moderate and strong correlations identified for both sets of cell populations analyzed; dashed lines indicate correlations identified for at least one set of cell populations (see Supplementary Tables 4 and 5). (**B**–**D**) Dendrograms for hierarchical clustering with Euclidean distances (B); the plots for k-means clustering, (k = 4) (**C**), and principal component analysis (PCA) (**D**), based on significant Spearman’s correlation coefficients (after FDR control using the Benjamini–Hochberg procedure) demonstrate similar cluster composition for both sets of cell populations analyzed: (top row) undifferentiated and differentiating ESCs, EGCs and ECCs and (low row) undifferentiated and differentiating ESCs, EGCs and ECCs and E 7.5 embryos. (**D**) PCA biplots of Spearman’s correlation coefficient datasets for *Mage* family and marker gene expression patterns. A PC1 and PC2 variances of 85% and 8.4–9.6%, respectively, indicate a clear separation of individual Mage expression patterns in the studied cell populations according to the involvement in self-renewal and differentiation (in correlations with respective gene markers). PCA outcomes are consistent with the results of cluster analyses (**B**–**D**). Colors indicate relative contributions of certain *Mage* genes in PC1 and PC2.

To clarify whether the expression of individual *Mage* genes can be associated with the self-renewal or differentiation of pluripotent and teratocarcinoma cells, the Spearman’s rank correlation coefficients (*rho)* were estimated to evaluate the dependence between the expression of individual *Mage* genes and marker genes in undifferentiated and differentiating ESCs, EGCs, ECCs and E7.5 embryos (Supplementary Tables 4 and 5). To evaluate the correlation significance, false discovery rate (FDR) correction was applied using the Benjamini–Hochberg procedure (*Q* = 0.25). On average, 41% of *Mage* family genes had moderate and strong correlations (negative and positive) with the expression of 6–9 marker genes, 18% of them had only 1–5 such correlations and 41% had no significant correlations with marker genes. The significant moderate and strong correlations (*rho ≥0.5* and *rho≤-0.5*) and possible relationships between the studied genes were summarized and visualized using the Graphviz software (Figure 6A).

In the next step, to discover potential functionally related sets of *Mage* genes, the hierarchical and *k*-means clustering as well as principal component analysis (PCA) based on the Spearman’s correlation coefficient data for the *Mages* and marker genes with FDR control were performed. The clustering analysis identified two *Mage* gene clusters, which can be regarded as functional gene groups because their expression strongly correlated with the expression of proliferation and differentiation gene markers (Figure 6B, 6C). These two clusters were identified regardless of the clustering algorithm used for both cell population sets (Figure 6B, 6C). The first common cluster includes *Mage-a4,* whose expression positively correlates with pluripotency and self-renewal marker gene expression and negatively correlates with the expression of differentiation markers. The second cluster includes six genes, *Mage-a8, Mage-b1, Mage-d1, Mage-d2, Mage-e1* and *Mage-l2,* whose expression strongly positively correlates with differentiation markers and negatively correlates with the expression of pluripotency and self-renewal markers (Figure 6B, 6C). Among the second cluster genes, the greatest expression dynamics are observed for ubiquitously expressed *Mages*. According to the estimated distances (Euclidian distance, Ward’s method) between the identified clusters and the opposite correlations with the expression of marker genes, the first and second clusters may be considered as functional antagonists. PCA also indicates a clear separation between the Mage gene groups, which belong to clusters 1 and 2 as identified by *k*-means clustering (Figure 6D). Moreover, *Mage-b16* was defined as an individual group (cluster 4) during *k*-means clustering (*k* = 4) and PCA and its expression was correlated with the expression of lineage markers, *Oct4* and *C-myc* but not with *Nanog* and *E-ras.*

Clustering and PCA outcomes for the other 9 *Mage* genes (*Mage-a2,* *Mage-a6, Mage-a10, Mage-b3-b5, Mage-b18, Mage-h1* and *Mage-e2)* were similar for two sets of cell populations (Figure 6; Supplementary Tables 4 and 5). No significant correlations after applying the Benjamini–Hochberg procedure were revealed for the 7 *Mage* genes of cluster 3; however, *Mage-a10* and *Pax6* expressions were correlated with expression of somatic lineages and *Mage-b18* expression was correlated negatively with the expression of the germ cell marker *Mvh.*

## DISCUSSION

To improve our understanding of the biological roles of CTAs in carcinogenesis, development, and differentiation, the dynamics of the expression patterns of *Mage* family members during the early differentiation of normal pluripotent stem cells and their malignant counterparts, teratocarcinoma cells, were verified using gene co-expression analysis. The clustering of gene expression patterns and analysis of correlations between the expression of *Mage* family members and proliferation and lineage differentiation markers revealed three main findings: (i) the similarity of the *Mage* gene expression patterns in differentiating ESCs and E 7.5 embryos; (ii) dissimilarities of the *Mage* family profiles in normal pluripotent stem (ESCs R1 and EGCs-10) and teratocarcinoma cells (ECCs F9 and P19); and (iii) strong correlations between the *Mage* expression patterns and differentiation states. These findings indicate that *Mage* expression in ESCs is not a phenomenon of rearranged gene expression in cultured pluripotent cells, but is involved in the regulation of lineage differentiation during the early development. However, aberrant *Mage* family expression patterns, like the patterns of marker genes, is probably the result of a general rearrangement of the regulatory network in teratocarcinoma cells with impaired differentiation potential (Figures 1D, 3 and 5). Therefore, the *Mage* family expression pattern can be considered as a biomarker panel for the validation of normal and abnormal stem cell differentiation. Thus, difference in the expression levels of *Mage-a2* detected in this work between mouse pluripotent and teratocarcinoma cells, likewise in *MAGE-A2* expression between human ESCs and ECCs [34], may be characteristic for teratocarcinoma cells and contribute to their malignant phenotype with imbalance of proliferation and differentiation potentials. Additionally, the over-expression of *MAGEA2* was found to intensify the self-renewal and to repress differentiation efficiency of human induced pluripotent stem cells while depletion of *Mageb16* in mouse ESCs induced more effective differentiation [49, 50]. In cancer cells, MAGEA2 and MAGEA6 promote cell growth and survival by targeting tumor suppressor protein AMP-activated protein kinase and preventing of ubiquitination and proteasome-dependent degradation, and acetylating p53 through histone deacetylase recruitment [6, 51–54]. Similarly, *MAGE-H1* can trigger apoptosis in melanoma cells through involvement of JNK/p38 pathway [55].

In our study, presumably functionally related gene modules were identified using the approaches of co-expression analyses (Spearman’s correlation analysis and clustering) for the expression patterns of *Mage* family genes and regulators of pluripotency, self-renewal and lineage differentiation in pluripotent stem and teratocarcinoma cells. We showed that the expression of *Mage-a4* (the first gene cluster) is strongly positively correlated with the expression of *C-myc* and *E-ras* and negatively correlated with lineage marker expression. Accordingly, *Mage-a4* expression appeared to be associated with the up-regulation of self-renewal and the down-regulation of differentiation of ESCs, EGCs and ECCs (Figure 6). This putative role of *Mage-a4* in pluripotent stem cells is supported by the findings of previous studies, which reported the influence of MAGE-A4 on cell cycle progression, proliferation and apoptosis in human cancer cells. MAGE-A4 can bind to Miz-1 and indirectly affect C-myc through regulation of the transcription of p21^CIP1^ and thereby influence the growth rate and p53-dependent and p-53-independent apoptosis in various cancer cells [2, 5, 7, 56, 57]. The overexpression of *MAGE-A4* promoted the growth of spontaneously transformed normal oral keratinocytes by inhibiting apoptosis and growth arrest in the G1-phase of the cell cycle [56]. Moreover, MAGE-A4 may be a direct target of TWIST1 [58], which also up-regulates cell cycle progression, proliferation and migration and inhibits cell death in cancer and embryonic cells [59].

Conversely, the expression of *Mage-a8, Mage-b1, Mage-d1, Mage-d2, Mage-e1* and *Mage-l2* (the second gene cluster) correlated negatively with the expression of self-renewal and pluripotency markers and positively with the expression of lineage differentiation markers (Figure 6). Consequently, the *Mage* genes in these two clusters may be considered as functional antagonists with opposing roles in the regulation of self-renewal and differentiation in mouse pluripotent stem and teratocarcinoma cells. Although all Mage family proteins of both classes contain the conserved Mage family domain (MHD), their N- and C-terminal regions differ significantly (Homologene, https://www.ncbi.nlm.nih.gov/homologene) [21, 41, 60]. Structural identity between the proteins of the first and second clusters was found in the range of 10–40%, except for Mage-a4 and Mage-a8 proteins (69%). In addition, Mage-d1, Mage-d2, Mage-e1 and Mage-l2 display 15–30% similarity of protein sequences. Such significant structural differences between members of the first and second clusters allow potential interactions with diverse domains in protein complexes activated during self-renewal and early differentiation. Our data are consistent with previous findings showing the involvement of the second cluster genes in the regulation of proliferation, apoptosis, migration and differentiation of cancer and embryonic cells. Thus, the expression of MAGE-D1 proteins was significantly reduced in human breast carcinoma cells compared to untransformed immortal mammary epithelial cell lines, while the overexpression of MAGE-D1 in hepatocarcinoma, osteosarcoma, breast carcinoma and kidney epithelial cell lines led to the suppression of cell migration, invasion, adhesion and to the arrest of cell proliferation at the G1/S and G2/M stages through a p53-dependent pathway [61–63]. Reportedly, both classes of MAGE family proteins can bind and activate RING E3 ubiquitin ligases and regulate p53 protein stability in cancer cells [64].

During development and differentiation, the expression of Mage-d1/NRAGE exhibited specific spatio-temporal patterns in the neural structures and differentiating osteogenic cells [65–67], and Mage-d1 and Mage-d2 exhibited distinct expression patterns in mouse embryonic tissues of neuroectodermal and mesodermal origin [20]. Predominant *MAGE-A8* expression was also detected in the early differentiating mesenchymal, neuroectodermal and extraendodermal cells derived from human ESCs [34], indicating a possible common mechanism with *MAGE-A8/Mage-a8* regulating the early differentiation stages of mouse and human pluripotent stem cells. *Mage-l2* has been shown to involve in neural development and the regulation of male and female reproductive functions [21, 68–70], while *Mage-b16* (cluster 4) expression was associated with the expression of genes regulating pluripotency, spermatogenesis and somatic lineage differentiation [50]. Noteworthy, the differential expression of both classes of Mage family genes was detected during development of reproductive and somatic derivatives in mice (Mouse Genome Informatics: references: J:262143; J:255000; J:257298; J:257299).

Obviously, the regulation of Mage family gene expression during the early development and carcinogenesis is associated with epigenetic mechanisms via DNA methylation and histone modification, as well as non-coding RNAs, miRNAs and ceRNA [71, 72]. During RA-induced differentiation of pluripotent and teratocarcinoma cells, all these epigenetic mechanisms can be recruited for initiation/repression of expression of regulatory and Mage family genes, like the miR-152-mediated effects on the genome-wide methylation state during RA-induced neuroblastoma cell differentiation [73]. MiR-874, miR-143-3p, miR-876-5 and miR-6775-3p have also been shown to contribute to the down-regulation of *MAGE* gene expression in cancer cells [74–77] and vice versa, MAGEH1 can suppress breast cancer metastases through upregulating mir-200a/b expression [78].

**Figure 7:**
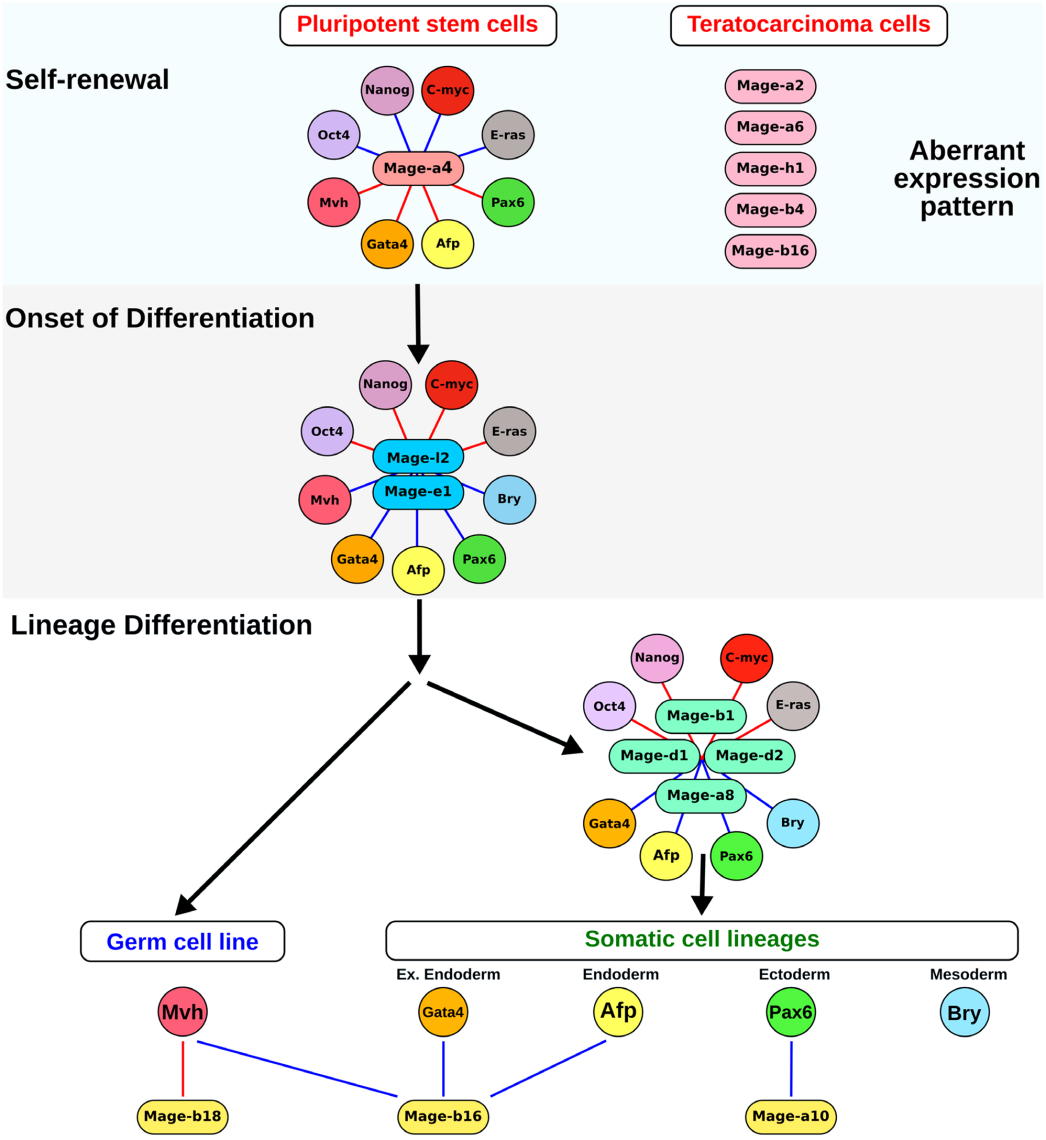
A model for *Mage* family involvement in the self-renewal and differentiation of pluripotent stem and teratocarcinoma cells. The first module with *Mage-a4* is involved in the positive regulation of self-renewal of pluripotent stem and teratocatcinoma cells. Additional module with variable expression of *Mage-a2, Mage-a6, Mage-h1, Mage-b4* and *Mage-b16* is associated with teratocarcinogenesis. The second module with *Mage-e1* and *Mage-l2* is activated after the initiation of differentiation. The early somatic lineage differentiation is associated with the expression of *Mage-a8, Mage-b1, Mage-d1* and *Mage-d2*, which belong to the third gene module. Additionally, the germ and somatic lineage differentiation correlates with changes in the expression of *Mage-b16,* *Mage-b18* and *Mage-a10*. Blue and red lines indicate positive and negative correlations, relatively.

To summarize the obtained data of gene co-expression analysis, a model of *Mage* family involvement in the regulation of different steps of self-renewal and differentiation of pluripotent stem and teratocarcinoma cells is presented in the scheme (Figure 7). Thus, several *Mage* gene modules were identified during normal and cancerous differentiation of pluripotent stem cells. The first predicted module includes *Mage-a4,* which contributes during the self-renewal stage. The gene module with variable expression of *Mage-a2, Mage-a6, Mage-b4, Mage-h1* and *Mage-b16* represents an additional teratocarcinoma-specific module activated during the self-renewal. The second module with *Mage-e1* and *Mage-l2* is involved in the regulation of the onset of differentiation of both somatic and germ cell precursors because the expression of these genes correlated positively with the expression of most lineage differentiation markers. The third module, which includes *Mage-d1, Mage-d2, Mage-a8* and *Mage-b1* is associated with the early differentiation of somatic lineages. In addition, the expression of *Mage-b16, Mage-b18* and *Mage-a10* correlates with a commitment to the certain germ and somatic lineages. In accordance to the present model, the Mage family proteins, involved in the regulation of proliferation and differentiation of embryonic and cancer cells, can play a role of protein hubs in the regulatory network and be important for carcinogenesis. Mage proteins, as potential intrinsically disordered proteins with a high binding plasticity, can interact with many partners and change their activity. In addition, Mage family proteins involved in preventing of protein degradation can affect the stability, time and level of expression of key regulators of cellular processes.

To conclude, the present study identified aberrant expression patterns for 5 of 17 (29%) *Mage* family genes along with 6 of 9 (67%) marker genes when comparing pluripotent stem and teratocarcinoma cells. The clustering analysis revealed strong correlations between *Mage* expression patterns and malignant and differentiation states. Clustering analysis identified two *Mage* clusters/modules as functional antagonists with opposing roles in the regulation of proliferation and differentiation of pluripotent stem and teratocarcinoma cells, and further investigations of predicted functional modules with the identified *Mage* gene clusters are needed to evaluate their contributions in the regulatory protein networks of normal and cancer stem cells.

## MATERIALS AND METHODS

### Cell culture

Mouse ESCs of the R1 line were kindly provided by Dr. A. Nagy (Mount Sinai Hospital, Toronto, Canada). Mouse EGC-10 cell line were kindly provided by Dr. A. McLaren (WTCR Institute of Cancer and Developmental Biology, Cambridge, UK). Mouse ECCs of the F9 and P19 lines were obtained from the Russian Cell Culture Collection (http://www.rccc.cytspb.rssi.ru/). The ESCs, EGCs and ECCs were cultivated in DMEM supplemented with 2 mM L-glutamine, 1% non-essential amino acids (all HyClone, GE Healthcare Life Sciences, USA), 0.1 mM β-mercaptoethanol (Sigma-Aldrich, USA) and 15% Characterized Fetal Bovine Serum (HyClone, USA). Undifferentiated mouse ESCs and EGCs were grown in gelatin-coated tissue culture plates in medium containing 10 ng/ml leukemia inhibitory factor (LIF, L-5158, Sigma-Aldrich).

To induce differentiation, the ESCs, EGCs and ECCs were seeded at a density of 5000 cells/cm^2^ and treated with 10^–6^ M all-trans-retinoic acid (RA, Sigma-Aldrich) for 5 or 10 days. The medium without LIF and supplemented with RA was changed daily. For microscopy analyses (fluorescent immunocytochemistry, alkaline phosphatase activity detection and cell labeling in the S-phase of the cell cycle), the undifferentiated cells (5000/cm^2^) were seeded in an 8-well Lab-Tek II chamber slide system plates and induced to differentiation with RA using the same schedule (Nalge Nunc International, USA). All experimental series were performed in triplicate. All cell lines were routinely tested using MycoFluor™ Mycoplasma Detection Kit (Invitrogen, USA).

### Mouse embryos sampling and ethics approval statement

Animal maintenance and experiments were approved by the Ethics Committee of Koltsov Institute of Developmental Biology of Russian Academy of Sciences and performed in accordance with the Russian Federation legislation (Order of the Ministry of Health and Social Development of the Russian Federation No 708n, August 28, 2010) based on the Directive 2010/63/EU of the European Parlament and of the Council on the protection of animals used for scientific purposes.

C57Bl/6 mice at the age of 2–3 months were obtained from the Animal Breeding Facility-Branch “Pushchino” (Institute of Bioorganic Chemistry, Russian Academy of Sciences). To obtain embryos, females were mated with males overnight, and vaginal plugs were tested following morning (embryonic stage E0.5). Fertilized females were sacrificed by cervical dislocation, and embryos at the E7.5 stage were recovered from the uterus. After the dissection of the embryos from the extraembryonic tissues the total RNAs were extracted from the isolated epiblasts and the adjacent extraembryonic endoderm.

### Alkaline phosphatase (ALP) activity detection

The cells were fixed with 2% paraformaldehyde in PBS, pH 7.0, for 15 min and incubated in a solution containing 10 ml 0.02 M Tris-HCl buffer pH 8.7, 1 mg Naphtol-AS-B1-phospate and 5 mg Fast Red dye (all from Sigma-Aldrich) at 37°C for 1 h.

### EdU-labeling of cells in the S-phase of the cell cycle

The labeling and detection of cells in the S-phase of the cell cycle were performed by incubating the cells with 10 μM 5-ethynyl-2-deoxyuridine (EdU) for 1 h and then with a reaction solution from the Click-iT^®^ EdU Imaging Kit with Azide-Alexa488 (C10083, Molecular Probes, USA) according to the protocol recommended by the manufacturer. After the completion of the Click-iT^®^ EdU reaction, the cells were fixed with 3% paraformaldehyde in PBS for further immunofluorescence staining.

### Immunofluorescent analysis

After treatment with 3% paraformaldehyde in PBS for 1 h, cells were washed with PBS, permeabilized with 0.5% Triton X-100 (Sigma-Aldrich) in PBS for 30 min and incubated in 3% Bovine Serum Albumin, Fraction V (BSA, Sigma-Aldrich) in PBS for 1h for nonspecific reaction blocking. The cells were incubated in a solution of primary antibodies in PBS with 0.25% Tween 20 (Sigma-Aldrich) at 4°C overnight. Primary rabbit anti-Mage (sc-10749), rabbit anti-Oct4 (sc-9081) and goat anti-Gata4 (sc-1237) antibodies (Santa Cruz Biotechnology, USA) were used at a dilution of 1:100. Secondary chicken anti-rabbit antibody conjugated with Alexa Fluor 594 (A-21442, Molecular Probes), and donkey anti-goat with Alexa Fluor 488 (A-11055, Molecular Probes) were diluted to 1:900 in PBS-Tween solution and applied to the cells for 3 h at room temperature. DAPI (Sigma-Aldrich) was applied for 20 min for nuclear staining. The preparations were mounted in a glycerol-based mounting medium (Ibidi, Germany) and examined under a Leica DMRXA2 fluorescent microscope. For negative controls, the primary antibodies were omitted, and the same staining procedure was used. For positive control, immunostaining of cryosections of the mouse testis was performed, as described previously [46]. Transverse cryosections were prepared from E7.5 mouse embryos fixed with 3% paraformaldehyde using the same immunostaining protocols.

### Flow cytometry

To analyze the cell cycle distributions, the cells were suspended in 0.05% Trypsin-EDTA (HyClone) and fixed with cold 70% ethanol. After triple washing with PBS the cells (10^6^/ml) were incubated in PBS containing 20 μg/mL of propidium iodide (Molecular Probes) and 200 μg/mL of RNAse A (EN0531, Fermentas, Lithuania) for 30 min. The probes were analyzed immediately after staining using a Cytomics FC500 flow cytometer (Beckman Coulter, USA) and MultiCycle AV Software (Phoenix Flow Systems, San Diego, CA, USA).

For the flow cytometry analysis of Oct4-expressing cells, the cells (10^6^/ml) were fixed with 3% paraformaldehyde in PBS for 15 min, washed with PBS and treated with 0.5% Triton X-100, 3% BSA and rabbit anti-Oct4 antibodies (1:200, sc-9081, Santa Cruz Biotechnology) in PBS for 40 min. After washing, the cells were incubated in PBS solution with 0.5% Triton X-100, 3% BSA and secondary chicken anti-rabbit antibodies conjugated with Alexa-488 (1:1000, A-21441, Molecular Probes) for 30 min. For the negative control, the cells were treated with normal rabbit IgG (sc-3888, Santa Cruz Biotechnology) and then with the same secondary antibody solution.

### Protein extractions and western blots

The cells were washed with ice cold PBS, and then lysed in 300 μl of NP-40 solution (0.5% Nonidet P-40, 50 mM Tris pH 8.1, 50 mM NaCl solution; all reagents from Sigma-Aldrich) with cOmplete™ ULTRA Protease Inhibitor Cocktail (05892970001; Roche-Sigma-Aldrich). Cell lysates were clarified by centrifugation at 14000 × g for 20 min at 4°C. Protein concentrations were determined using NanoDrop 2000 (Thermo Scientific, USA) and the BCA Protein Assay Kit (23227; Pierce-Thermo Scientific, USA) according to the manufacturer’s instructions. The samples were diluted in 2× Laemmli buffer (Sigma-Aldrich) and denatured by boiling followed by chilling on ice.

Probes containing 7 μg of proteins were separated by SDS-PAGE in 10% gels (A2792; Sigma-Aldrich) at 150 V for 1.5 h and then transferred to nitrocellulose membrane (RPN303D; Amersham/GE Healthcare Life Sciences, USA) at 120 mA for 2 h using the Mini Trans-Blot cell (Bio-Rad Laboratories, USA). Membranes were blocked in TBST (1×Tris-buffered saline, pH 7.5, 0.1% (vol/vol) Tween-20 (Sigma-Aldrich)) with 5% (wt/vol) nonfat dry milk (170-6404; Bio-Rad Laboratories) and immunoblotted with the following antibodies and dilutions overnight at 4°C: rabbit anti–MAGED2/D1 polyclonal antibody (NBP2-24694, lot AB 041309A-01; Novus Biologicals, USA; at 5 μg/ml), rabbit anti-Oct4 polyclonal antibody (sc-9081, Santa Cruz Biotechnology; at 5 μg/ml), goat anti-Gata4 polyclonal antibody (sc-1237; Santa Cruz Biotechnology; at 5 μg/ml), rabbit anti-Ddx4/Mvh polyclonal antibody (ab13840; lot 773844; Abcam, UK; at 2 μg/ml), mouse anti-α-tubulin monoclonal antibody (T-6793, Sigma-Aldrich; 1:2000). Membranes were washed 3 times for 20 minutes in TBST and incubated with the appropriate horseradish peroxidase-conjugated secondary antibodies: goat anti-rabbit and anti-mouse IgG (170-5046 and170-5047, respectively; Bio-Rad) and bovine anti-goat IgG (805-035-180; Jackson ImmunoResearch Laboratories, West Grove, PA, USA) at 1:5000 dilutions for 1 h at room temperature. After washing (3 times for 20 min), the chemiluminescent signals on the membranes were detected using the ECL Plus Western Blotting Detection Reagents (RPN 2132; Amersham/GE Healthcare Life Sciences) and captured using Fusion Solo S chemiluminescence imaging system (Vilber Lourmat, France). The relative band intensity was quantified using the Gel Analysis method outlined in the ImageJ documentation (http://rsb.info.nih.gov/ij/docs/menus/analyze.html#gels). Pre-stained molecular weight protein standard SDS-PAGE Broad Range Standard (161-0318, Bio-Rad) was used.

### RNA extraction and cDNA synthesis

Total RNAs were extracted from the undifferentiated and differentiated ESCs, EGCs, ECCs (0.5 × 10^6^ cells per sample) and E7.5 mouse embryos (*n* = 3 per samples) using the TRIzol^®^ Reagent (15596-018, Invitrogen). Each sample was treated with TURBO DNase (AM1907, Ambion/Invitrogen, USA) according to the manufacturer’s recommendations. The RNA yield and quality were analyzed using NanoDrop 2000 (Thermo Scientific, USA). cDNAs were synthesized using oligo-dT18 primer (Fermentas) and SuperScript III reverse transcriptase (Invitrogen) according to the manufacturer’s protocols. Two micrograms of total RNA were used for cDNA synthesis. Total RNAs extracted from adult mouse testes were used as a positive control.

### Quantitative real-time polymerase chain reaction (qRT-PCR)

A quantitative analysis of gene expression was carried out using the Applied Biosystems 7500 Real-Time PCR System (Life Technologies, USA) with the qRT-PCR master mix with EVA Green stain and ROX passive reference dye (Sintol, Russia). The following protocol was used: denaturation at 95°C for 5 min, followed by 40 cycles at 95°C for 15 sec and at 60°C for 1 min. All experiments were run in triplicate. The expression levels of target mRNAs were normalized to the expression of the reference gene hypoxanthine-guanine phosphoribosyltransferase (*Hprt*). The relative levels of target gene expression were calculated using the comparative 2-^ΔΔCt^ method (ABI Relative Quantification Study software, Applied Biosystems, USA). Specific primers were designed based on GenBank and Ensemble data concerning the annotated sequences of the target genes using the Beacon Designer 8.0 software (Premier Biosoft, Palo Alto, CA, USA) (Supplementary Table 6). Due to high homology of mouse *Mage* genes, the primers were designed to detect the expression of five of eight genes the *Mage-a* subfamily and all genes of the *Mage-b,* *Mage-d*, *Mage-e, Mage-l* and *Mage-h* subfamilies The designed Mage primers were pre-screened using cDNAs synthesized from total RNAs from adult mouse testes (Supplementary Figure 4 and Supplementary Table 7).

### Statistical and bioinformatics analyses of gene expression

The gene expression data were subjected to statistical analysis using the R v.3.2.3 software (http://www.r-project.org). The averaged ΔCt values (Supplementary Tables 1–3) for target genes obtained from three independent experiments (*n* = 3) were used for statistical analysis using one-way ANOVA followed by a Tukey post-hoc test.

To determine the similarity/dissimilarity of the gene expression patterns of the studied cells and embryos, the heatmaps with the hierarchical dendrograms were constructed using hierarchical clustering algorithm with Euclidean distances measurement based on the averaged ΔCt values of studied genes after data scaling using the R v.3.2.3 software.

The averaged ΔCt values for *Mage* and marker genes were used in the linear regression analysis based on Spearman’s correlation method (Supplementary Tables 4 and 5). The normality of the ΔCt values for each gene pair was evaluated using the Shapiro-Wilkoxon normality test. Correlations were considered as strong at 0.7<rho<0.9, moderate at 0.5<rho<0.69, and weak at 0.3<rho<0.49. Significant correlations between the expression levels of *Mage* and marker genes were calculated using the Benjamini–Hochberg procedure for FDR control (*Q* = 0.25). Visualization of gene co-expression network for the strong and moderate (rho ≤-0.5 and rho ≥0.5) correlations between the expression of *Mage* and marker genes was performed using the Graphviz software (http://www.graphviz.org).

To discover *Mage* gene modules involved in the regulation of proliferation and differentiation, the hierarchical and k-means clustering, as well as principal component analysis (PCA) based on Spearman’s correlation coefficient data were performed after the Benjamini–Hochberg procedure (*Q* = 0.25) using the R v.3.2.3 software. The insignificant Spearman’s correlation coefficients (after Benjamini–Hochberg procedure) were taken as 0 in the data matrices. In hierarchical clustering, the measurement of the Euclidean distances was carried out by the Ward method. For the k-means clustering, the cluster composition was analyzed for k = 4.

## SUPPLEMENTARY MATERIALS


