# Supplementary material for: Expression dynamics of Mage family genes during self-renewal and differentiation of mouse pluripotent stem and teratocarcinoma cells
Source: Oncotarget. 2019 May 14;10(35):3248–66. doi: 10.18632/oncotarget.26933 (PMC6524934; doi:10.18632/oncotarget.26933)
Supplement: Supplementary file 1 [file oncotarget-10-3248-s001.pdf]

Expression dynamics of *Mage* family genes during self-renewal and differentiation of mouse pluripotent stem and teratocarcinoma cells

SUPPLEMENTARY MATERIALS

Supplementary Table 1: Averaged ΔCt/SD values for qRT-PCR analysis of gene expression in undifferentiated ESC R1, EGC-10, ECC F9, ECCP19

	ESC R1		EGC-10		ECC F9		ECC P19	
	ΔCt	SD	ΔCt	SD	ΔCt	SD	ΔCt	SD
Oct4	–3.501167	0.203673	–4.063395	0.284517	–3.755070	0.177018	–4.586677	0.184608
Nanog	–0.000610	0.133213	–0.084123	0.156496	2.128930	0.158934	2.378477	0.126882
C–myc	–0.447248	0.256809	–0.562806	0.285848	–0.158731	0.243538	–1.004229	0.120331
E–ras	1.361930	0.137376	1.807971	0.153228	1.145161	0.234649	1.699921	0.239407
Mvh	2.128251	0.037315	2.443063	0.160889	3.163674	0.251501	7.008224	0.163313
Gata4	5.372614	0.203338	6.234786	0.221450	3.668681	0.095056	6.299318	0.269996
Afp	9.374775	0.211126	10.144185	0.181806	9.213038	0.099342	9.396228	0.265403
Pax6	7.419222	0.110000	7.962599	0.360344	7.190369	0.096166	5.802730	0.102140
Bry	9.042114	0.029163	8.886931	0.216844	9.292353	0.163933	7.851796	0.153857
Magea2	8.690732	0.146359	9.686414	0.287232	6.852456	0.175001	10.133675	0.192499
Magea4	8.425477	0.15475118	8.345166	0.126564	8.765286	0.161985	8.339009	0.160059
Magea6	10.613347	0.2454554	10.625308	0.337628	6.412736	0.224452	10.201073	0.191974
Magea8	10.940110	0.27004924	9.867871	0.244881	10.999196	0.101723	9.762673	0.261965
Magea10	10.172827	0.25607431	10.377820	0.309298	9.657189	0.282922	9.439465	0.182793
Mageb1	10.582868	0.20030234	11.362831	0.280000	10.257787	0.194570	10.350467	0.114747
Mageb3	8.559841	0.18016933	8.090735	0.162360	8.204238	0.178394	8.703820	0.235775
Mageb4	8.873193	0.16132326	9.965228	1.061631	9.423430	0.139274	10.370410	0.281959
Mageb5	12.418937	0.3641862	11.711190	0.438565	12.151481	0.139274	11.482739	0.392320
Mageb16	5.322598	0.1599533	6.044364	0.249601	4.916920	0.168409	8.438762	0.158866
Mageb18	8.786191	0.3605847	7.522509	0.356739	8.158746	0.382037	7.699327	0.224916
Maged1	–1.135971	0.15395792	–1.843499	0.254395	–2.190240	0.389713	–1.558423	0.114153
Maged2	2.465937	0.09073673	2.383796	0.133781	2.552799	0.129982	2.491382	0.131757
Magee1	4.727014	0.22486207	3.754762	0.178922	4.926140	0.239058	4.939393	0.154646
Magee2	6.975409	0.17364967	8.822598	0.253456	6.492977	0.253545	7.926259	0.332247
Mageh1	9.535243	0.16009492	8.550313	0.344253	7.590039	0.245494	6.334561	0.181904
Magel2	8.768645	0.17481035	8.381588	0.169360	8.462224	0.158723	9.350467	0.114747

Supplementary Table 2: Averaged ΔCt/SD values for qRT-PCR analysis of gene expression in differentiating ESC R1, EGC-10, ECC F9, ECCP19 after 5day RA stimulation

	ESC R1 RA5		EGC-10 RA5		ECC F9 RA5		ECC P19 RA5	
	ΔCt	SD	ΔCt	SD	ΔCt	SD	ΔCt	SD
Oct4	2.203574	0.152391	2.410980	0.103040	0.189378	0.119404	–0.788572	0.081245
Nanog	2.824704	0.054165	3.258162	0.163081	6.500351	0.118527	8.171848	0.164228
C-myc	3.384322	0.124961	1.180004	0.134626	1.080395	0.066036	1.105616	0.186992
E-ras	5.534152	0.096085	4.777456	0.070672	2.834838	0.064890	6.835654	0.091986
Mvh	–0.255645	0.03640826	1.050925	0.217054	1.393271	0.196795	6.301350	0.136110
Gata4	–0.469282	0.130564	3.257800	0.003112	–0.696892	0.133431	2.596374	0.112996
Afp	5.533659	0.139814	8.361759	0.172380	5.398602	0.133012	6.301350	0.244376
Pax6	4.370574	0.120031	3.034269	0.143296	4.040314	0.116495	3.264228	0.076637
Bry	7.375867	0.091240	7.946080	0.208521	8.085884	0.136048	8.085884	0.136048
Magea2	8.819748	0.251657	8.796891	0.266242	8.977286	0.190763	9.829566	0.189580
Magea4	10.017493	0.165013	9.736087	0.148868	9.700796	0.147089	8.873096	0.270847
Magea6	9.825872	0.250395	9.801722	0.241685	8.431954	0.232348	9.975919	0.377228
Magea8	7.285315	0.160038	7.010790	0.139328	8.483275	0.174499	6.287233	0.119673
Magea10	10.623000	0.195705	10.570299	0.212100	9.329679	0.038338	9.346442	0.118871
Mageb1	10.000110	0.180022	10.050132	0.170110	9.562469	0.348604	9.993285	0.198723
Mageb3	8.293883	0.124175	8.133249	0.132142	8.805661	0.212839	8.589783	0.066835
Mageb4	9.807559	0.156080	10.526092	0.274986	9.011398	0.581439	10.076642	0.589137
Mageb5	11.931365	0.220159	12.638050	0.278455	11.209222	0.312661	11.167120	0.395847
Mageb16	3.937146	0.198536	4.652052	0.145656	3.741940	0.094932	7.736084	0.156964
Mageb18	10.666554	0.380909	9.745126	0.421512	9.507064	0.444161	5.990621	0.283556
Maged1	–4.486670	0.035804	–4.191296	0.125680	–5.089004	0.113269	–4.968672	0.019633
Maged2	–1.398219	0.038922	–0.464857	0.132855	–0.699048	0.146293	–2.203288	0.122260
Magee1	1.145931	0.132888	1.761934	0.149525	2.587044	0.173532	1.320625	0.125349
Magee2	6.542890	0.160580	8.222401	0.052334	6.509256	0.317981	6.142046	0.215327
Mageh1	8.921985	0.167424	7.299719	0.273876	5.400488	0.125008	5.291112	0.235023
Magel2	5.503847	0.108579	6.326383	0.130073	7.152322	0.212524	6.138818	0.154800

Supplementary Table 3: Averaged ΔCt/SD values for qRT-PCR analysis of gene expression in embryos at E7.5 stage, in ESCs differentiated spontaneously (ESC R1 -RA5) and after 10 day RA stimulation (ESRR1+RA10)

	E 7.5		ESC R1 -RA5		ESC R1 RA10	
	**ΔCt**	**SD**	**ΔCt**	**SD**	**ΔCt**	**SD**
Oct4	2.361820	0.024866	–0.869215	0.223437	1.715577	0.412705
Nanog	5.584545	0.234542	–0.370477	0.015287	4.741663	0.403897
C-myc	2.243361	0.040414	–0.337860	0.122273	1.215071	0.242381
E-ras	5.471590	0.268411	1.982622	0.154874	4.689236	0.406382
Mvh	6.030491	0.321303	6.419222	0.110000	5.367906	0.042543
Gata4	7.863339	0.343888	7.907380	0.286663	3.546959	0.353094
Afp	5.409803	0.098237	9.747573	0.198763	8.351289	0.285500
Pax6	5.930327	0.295194	7.922188	0.110000	4.111134	0.232950
Bry	8.565045	0.207707	8.613985	0.043485	8.535647	0.577582
Magea2	9.051693	0.044836	10.843139	0.397595	8.107572	0.215192
Magea4	9.234743	0.289523	7.402407	0.076058	10.816041	0.165212
Magea6	8.971819	0.194903	8.768933	0.485555	6.988190	0.170822
Magea8	8.989324	0.067541	12.845535	0.300806	9.330039	0.369134
Magea10	7.940138	0.101981	7.203930	0.337852	7.979294	0.173948
Mageb1	8.762777	0.203152	11.217374	0.337853	13.716409	0.331519
Mageb3	7.045881	0.205805	9.071439	0.286581	9.288148	0.261568
Mageb4	7.713755	0.202480	8.073211	0.361602	10.302490	0.178392
Mageb5	9.598783	0.055842	9.645192	0.288709	7.350061	0.213312
Mageb16	8.310954	0.144109	9.647298	0.388709	3.440945	0.145167
Mageb18	7.611266	0.078882	11.723844	0.166177	8.962530	0.148778
Maged1	–3.798262	0.150484	–0.856963	0.046198	–1.620576	0.160603
Maged2	2.361820	0.044866	3.003909	0.143660	0.277825	0.273222
Magee1	2.627139	0.119151	6.143772	0.073398	2.377417	0.142671
Magee2	6.837651	0.135707	7.184377	0.073398	9.482566	0.186242
Mageh1	9.835856	0.088213	9.314681	0.194199	9.774984	0.060384
Magel2	7.062494	0.089461	9.685489	0.130943	7.505463	0.099890

Supplementary Table 4: Spearman's rank correlation between the expression levels of *Mage* and marker genes in undifferentiated and differentiating ESCs R1, EGCs-10, ECCs F9 and ECCs P19

		Oct4	Nanog	C-myc	E-ras	Mvh	Gata4	Afp	Pax6	Bry
Magea2	*rho*	-0.3333	0.2857	-0.2857	0.4286	0.4762	0.1667	0.119	-0.1429	-0.503
	p	0.4279	0.5008	0.5008	0.2992	0.2431	0.7033	0.793	0.752	0.2039
Magea4	*rho*	0.9524	0.619	0.9762	0.6429	-0.7857	-0.881	-0.8571	-0.6905	-0.3952
	p	0.0011	0.115	4,0E-04	0.0962	0.0279	0.0072	0.0107	0.0694	0.3325
Magea6	*rho*	-0.4762	-0.5	-0.5238	-0.0238	0.2619	0.619	0.6667	0.5238	-0.012
	p	0.2431	0.2162	0.1966	0.9768	0.5364	0.115	0.0831	0.1966	0.9775
Magea8	*rho*	-0.6429	-0.8333	-0.6667	-0.9524	0.2381	0.5476	0.5714	0.8571	0.6826
	p	0.0962	0.0154	0.0831	0.0011	0.5821	0.171	0.1511	0.0107	0.0621
Magea10	*rho*	0.3095	-0.4048	0.3333	0.0952	-0.5952	0.119	0.1905	0.8571	-0.2515
	p	0.4618	0.3268	0.4279	0.8401	0.1323	0.793	0.6646	0.0107	0.5479
Mageb1	*rho*	-0.6667	-0.9286	-0.7143	-0.6429	0.2857	0.9048	0.9524	0.8095	0.3713
	p	0.0831	0.0022	0.0576	0.0962	0.5008	0.0046	0.0011	0.0218	0.3652
Mageb3	*rho*	-0.0476	0.5238	-0.0952	0.0952	0.2619	-0.2857	-0.4286	-0.2857	-0.2395
	p	0.9349	0.1966	0.8401	0.8401	0.5364	0.5008	0.2992	0.5008	0.5678
Mageb4	*rho*	0.0476	0.2857	0.0952	0.4286	0.2143	0.2381	0.1905	-0.4762	-0.5509
	p	0.9349	0.5008	0.8401	0.2992	0.6191	0.5821	0.6646	0.2431	0.157
Mageb5	*rho*	0.2619	-0.4524	0.1429	-0.381	-0.4762	0.2143	0.2381	0.1429	0.1796
	p	0.5364	0.2675	0.752	0.3599	0.2431	0.6191	0.5821	0.752	0.6703
Mageb16	*rho*	-0.7381	-0.2619	-0.6429	-0.1667	0.8571	0.7619	0.7143	0.3095	0.0958
	p	0.0458	0.5364	0.0962	0.7033	0.0107	0.0368	0.0576	0.4618	0.8215
Mageb18	*rho*	0.7143	0.1429	0.5952	0.119	-0.881	-0.5	-0.5238	-0.3095	-0.3952
	p	0.0576	0.752	0.1323	0.793	0.0072	0.2162	0.1966	0.4618	0.3325
Maged1	*rho*	-0.6429	-0.8333	-0.7143	-0.7143	0.3333	0.9048	0.881	0.7143	0.2994
	p	0.0962	0.0154	0.0576	0.0576	0.4279	0.0046	0.0072	0.0576	0.4713
Maged2	*rho*	-0.6667	-0.7143	-0.7143	-0.9524	0.4048	0.7381	0.6905	0.619	0.4791
	p	0.0831	0.0576	0.0576	0.0011	0.3268	0.0458	0.0694	0.115	0.2297
Magee1	*rho*	-0.8095	-0.5952	-0.881	-0.9048	0.619	0.7619	0.6905	0.5952	0.4551
	p	0.0218	0.1323	0.0072	0.0046	0.115	0.0368	0.0694	0.1323	0.2572
Magee2	*rho*	-0.1667	-0.5476	-0.3571	-0.1667	-0.1905	0.5714	0.619	0.3095	-0.1916
	p	0.7033	0.171	0.3894	0.7033	0.6646	0.1511	0.115	0.4618	0.6494
Mageh1	*rho*	-0.0476	-0.7619	-0.0714	-0.4048	-0.4286	0.2857	0.3571	0.6429	0.2036
	p	0.9349	0.0368	0.882	0.3268	0.2992	0.5008	0.3894	0.0962	0.6287
Magel2	*rho*	-0.7857	-0.619	-0.9048	-0.881	0.5714	0.7857	0.7143	0.619	0.4311
	p	0.0279	0.115	0.0046	0.0072	0.1511	0.0279	0.0576	0.115	0.2862

(before the Benjamini–Hochberg procedure for FDR control).

Supplementary Table 5: Spearman's rank correlation between the expression levels of *Mage* and marker genes in undifferentiated and differentiating ESCs R1, EGCs-10, ECCs F9 and ECCs P19 and E7.5 embryos

		Oct4	Nanog	C-myc	E-ras	Mvh	Gata4	Afp	Pax6	Bry
Magea2	*rho*	–0.3	0.2833	–0.2333	0.4	0.6	0.2667	0.1	–0.1333	–0.4268
	p	0.4366	0.463	0.5517	0.2912	0.0968	0.4933	0.81	0.7435	0.252
Magea4	*rho*	0.9	0.5833	0.9167	0.6333	–0.7	–0.65	–0.8	–0.65	–0.4352
	p	0.002	0.108	0.0013	0.076	0.0433	0.0666	0.0138	0.0666	0.2418
Magea6	*rho*	–0.4666	–0.4833	–0.4833	–0.0333	0.15	0.3333	0.6833	0.3667	–0.0669
	p	0.2125	0.1938	0.1938	0.9484	0.7081	0.3853	0.0503	0.3363	0.8641
Magea8	*rho*	–0.6333	–0.8	–0.6333	–0.9	0.2	0.5167	0.55	0.8833	0.7113
	p	0.076	0.0138	0.076	0.002	0.6134	0.1618	0.1328	0.0031	0.0317
Magea10	*rho*	0.0333	–0.5333	0.0667	–0.0667	–0.6333	–0.2167	0.4167	0.05	–0.2259
	p	0.9484	0.1475	0.8801	0.8801	0.076	0.5809	0.2696	0.9116	0.5588
Mageb1	*rho*	–0.7	–0.9	–0.75	–0.6667	0.0333	0.3333	0.95	0.6	0.2929
	p	0.0432	0.0020	0.0255	0.0589	0.9484	0.3853	4,00E-04	0.0968	0.4444
Mageb3	*rho*	–0.3	0.3	–0.3	–0.0667	0.1	–0.5	–0.1333	–0.35	–0.318
	p	0.4366	0.4366	0.4366	0.8801	0.81	0.1777	0.7435	0.3586	0.4043
Mageb4	*rho*	–0.1333	0.0333	–0.1667	0.1667	0.0667	–0.1333	0.4	–0.4833	–0.5356
	p	0.7435	0.9484	0.6777	0.6777	0.8801	0.7435	0.2912	0.1938	0.1373
Mageb5	*rho*	0.01666	–0.5666	–0.1167	–0.4667	–0.5667	–0.15	0.4333	0.0833	0.1088
	p	0.9816	0.1206	0.7756	0.2125	0.1206	0.7081	0.2499	0.8432	0.7806
Mageb16	*rho*	–0.4333	–0.1	–0.3833	–0.0333	0.8833	0.8167	0.4333	0.3	0.1004
	p	0.2499	0.81	0.3125	0.9484	0.0031	0.0108	0.2499	0.4366	0.7971
Mageb18	*rho*	0.4666	–0.0166	0.3833	–0.05	–0.8333	–0.5167	–0.3167	–0.3167	–0.3849
	p	0.2125	0.9816	0.3125	0.9116	0.0083	0.1618	0.4101	0.4101	0.3063
Maged1	*rho*	–0.6	–0.8333	–0.6667	–0.7167	0.3167	0.7667	0.8167	0.75	0.41
	p	0.0968	0.0082	0.0589	0.0369	0.4101	0.0214	0.0108	0.0255	0.273
Maged2	*rho*	–0.6166	–0.7333	–0.6833	–0.9167	0.35	0.65	0.65	0.6833	0.5356
	p	0.0857	0.0311	0.0503	0.0013	0.3586	0.0666	0.0666	0.0503	0.1373
Magee1	*rho*	–0.7333	–0.6166	–0.8167	–0.8833	0.5333	0.6667	0.6333	0.65	0.5021
	p	0.0311	0.0857	0.0108	0.0031	0.1475	0.0589	0.076	0.0666	0.1684
Magee2	*rho*	–0.1166	–0.5666	–0.35	–0.25	–0.15	0.5167	0.5833	0.3	–0.1004
	p	0.7756	0.1206	0.3586	0.5206	0.7081	0.1618	0.108	0.4366	0.7971
Mageh1	*rho*	0.1833	–0.4833	0.2167	–0.15	–0.2333	0.5	0.0667	0.6167	0.3096
	p	0.6436	0.1938	0.5809	0.7081	0.5517	0.1777	0.8801	0.0857	0.4175
Magel2	*rho*	–0.75	–0.6166	–0.8833	–0.9	0.4333	0.55	0.6333	0.6167	0.4603
	p	0.0255	0.0857	0.0031	0.002	0.2499	0.1328	0.076	0.0857	0.2125

(before the Benjamini–Hochberg procedure for FDR control).

Supplementary Table 6: Real-time reverse transcription polymerase chain reaction (qRT-PCR) primers

Gene	Accession no.	Primer sequences	Amplicon (bp)
Hprt	NM_013556.2	5′ ttgggcttacctcactgctttc 3′<br>5′ ctaatcacgacgctgggactg 3′	125
Oct4	NM_013633.2	5′ caccctgggcgttctctttg 3′<br>5′ gttctcattgttgtcggcttcc 3′	142
Nanog	NM_028016	5′ aactctcctccattctgaacctga 3′<br>5′ ggtgctgagcccttctgaatc 3′	136
Mvh	NM_010029.2<br>NM_001145885.1	5′ aggaatgccatcaaaggaacaac 3′<br>5′ gcccaacagcgacaaacaag 3′	119
Gata4	NM_008092	5′ tctcactatgggcacagcag 3′<br>5′ gggacagcttcagagcagac 3′	100
Afp	NM_007423.4	5′ cttccctcatcctcctgctac 3′<br>5′ acattcttctccgtcacgca 3′	109
Pax6	NM_013627.6	5′ taccagtgtctaccagccaatcc 3′<br>5′ gcacgagtatgaggaggtctga 3′	193
Bry	NM_009309.2	5′ atgctgcctgtgagtcataac 3′<br>5′ cgtgtgcgtcagtggtgtg 3′	177
C-myc	NM_001177352<br>NM_010849.4	5′ tgatgtggtgtctgtggagaaga 3′<br>5′ gcgtagttgtgctggtgagt 3′	144
E-ras	NM_181548.2	5′ gcctacaaagtctagcatcttgg 3′<br>5′ cgaagcattggtgagtcatctg 3′	191
Mage-a2	NM_020016.1	5′atcgtatctggagactttgtggac 3′<br>5′gggacctaaactggcactgc 3′	92
Mage-a4	NM_020280.2	5′ ctggtctctggcattggcat 3′<br>5′ cctgtcttgggcttactctgaac 3′	122
Mage-a6	NM_020019.3	5′ aaggcttggatcttgagcagat 3′<br>5′ cttacctggggttagaagggaaa 3′	80
Mage-a8	NM_020020.4	5′ ttgagatatagaggctgaaccttcca 3′<br>5′ ggcgaactcctttccaagactc 3′	88
Mage-a10	NM_001085506.1	5′ agcagagagagccacacct 3′<br>5′ gacccagagaccttgagtcct 3′	159
Mage-b1	NM_010759.1	5′ aggtctccattaagtccaaggtattc 3′<br>5′ ggaatctggaaggataagaatgacaac 3′	82
Mage-b3	NM_008545.2	5′ cctgttgcccttggacctatg 3′<br>5′ gcgtttcagcatcaagaagattaag 3′	168
Mage-b4	NM_001033492.2	5′ gggaatttcgcttagcaatcaagg 3′<br>5′ gtggcaagagacagcagatagg 3′	159
Mage-b5	NM_028847.1	5′ ggagaatcatccacttctgaagag 3′<br>5′ ggttgcggtggtgcttattc 3′	86
Mage-b16	NM_001113734.1	5′ accagccaatagccaatagtga 3′<br>5′ tcttcttcaatcagtgcctcttca 3′	166
Mage-b18	NM_173783.3	5′ tgagacaacttcacaagtcattgc 3′<br>5′ tcacgggcacggagtttg 3′	196
Mage-d1	NM_019791.2	5′ ttcattgcagaggttcagaagaga 3′<br>5′ agcatccagagcatccaagg 3′	90
Mage-d2	NM_001199246.1	5′ gctcgctctcagggcaaa 3′<br>5′ aagttctgggtcacggtcaa 3′	77
Mage-e1	NM_053201.4	5′ aagattagagagcaaggcaagga 3′<br>5′ gcgagcagcacgattcag 3′	148
Mage-e2	NM_053206.2	5′ ggaggctgtggaagatgagg 3′<br>5′ cgtcagatggaaccgaagaaga 3′	75
Mage-h1	NM_023788.3	5′ gctggcgtttgcggttca 3′<br>5′ gctcttgcgattgttgcgattc 3′	184
Mage-l2	NM_013779.2	5′ ccacacttacatcatcgtcaacaa 3′<br>5′ aggctcaagaccaccatcag 3′	105

Supplementary Table 7: Averaged ∆Ct/SD values for qRT-PCR analysis of *Mage* gene expression in adult mouse testes

	Testes	
	**∆Ct**	**SD**
Magea2	3.107270	0.091978
Magea4	4.057505	0.089887
Magea6	2.053839	0.092979
Magea8	3.828487	0.178280
Magea10	3.791387	0.145542
Mageb1	6.861729	0.171545
Mageb3	2.852348	0.158815
Mageb4	1.891993	0.159100
Mageb5	0.688219	0.197308
Mageb16	3.819262	0.194678
Mageb18	5.063253	0.143639
Maged1	-2.277032	0.110427
Maged2	0.278460	0.082640
Magee1	3.299875	0.146576
Magee2	10.16469	0.11107
Mageh1	8.181413	0.237694
Magel2	9.549878	0.194421

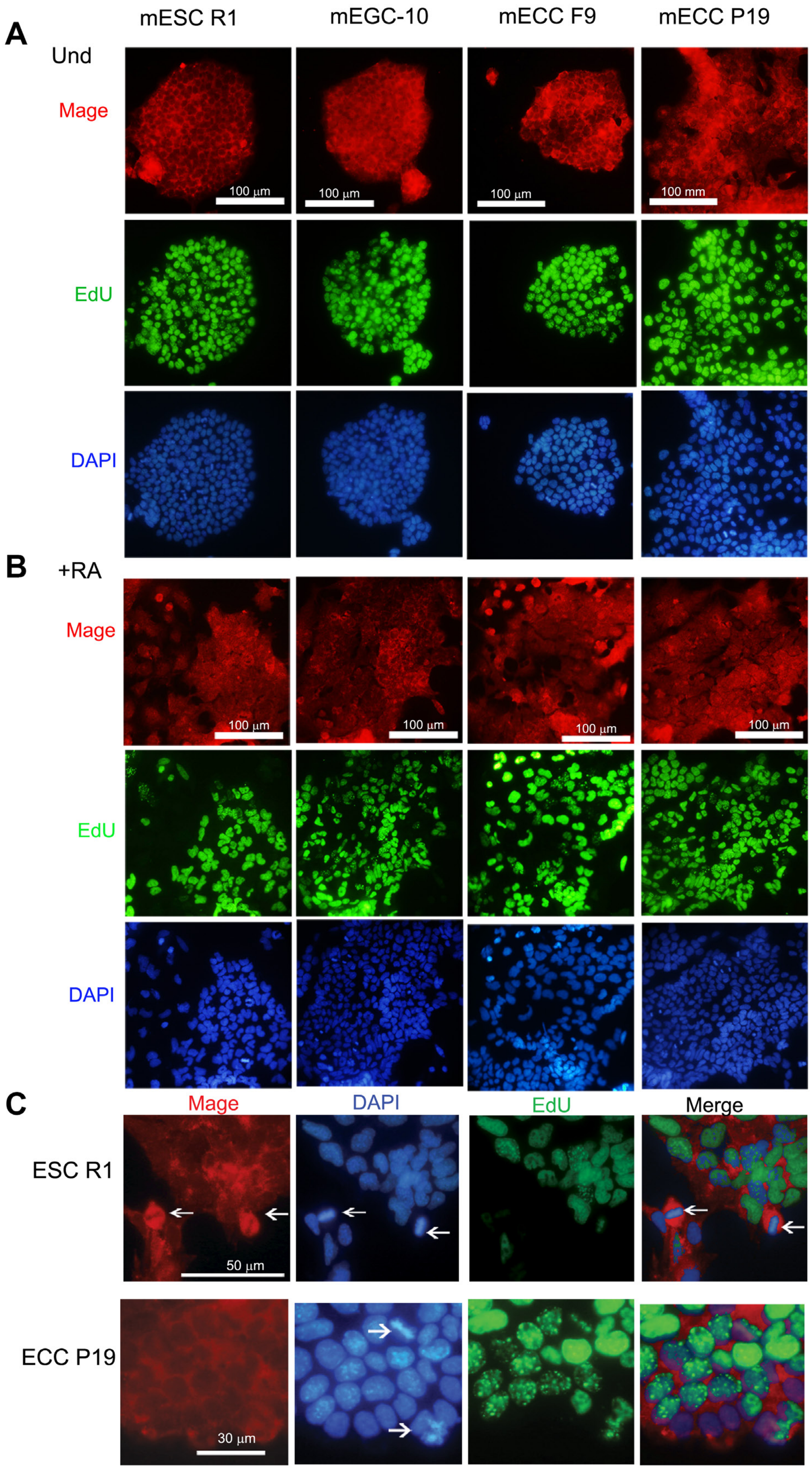

Supplementary Figure 1: Expression of the Mage proteins in undifferentiated and differentiated ESCs R1, EGCs-10, ECCs F9 and ECCs P19. Triple labeling (Mage+EdU+ DAPI) of undifferentiated (**A**) and differentiating (**B**) ESCs, EGCs and ECCs. Scale bar = 100 µm. (**C**) High magnification of the triple labeling (Mage+EdU+DAPI) of undifferentiated and differentiated ESCs. The arrows point to cells in the M-phase of the cell cycle that are expressing Mage proteins. Double-stained (Mage+DAPI) cells were in the G1- or G2-phases. Triple-stained (Mage+EdU+DAPI) cells were in the S- and G2-phases of the cell cycle. Scale bar = 50 µm.

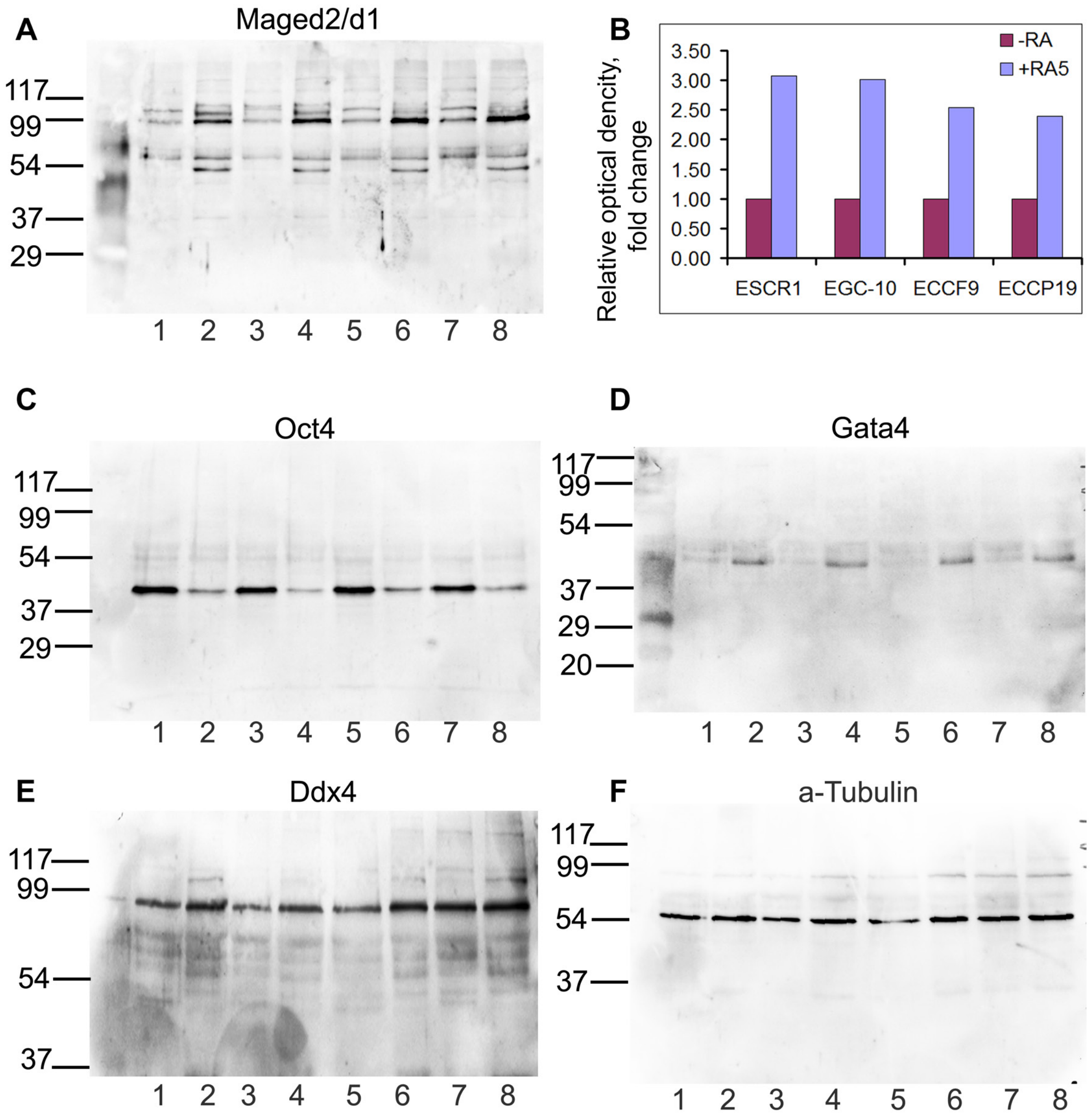

Supplementary Figure 2: Expression of the Mage-d1/Mage-d2 (**A**, **B**), Oct4 (**C**), Gata4 (**D**), Ddx4 (**E**) and a-Tubulin (**F**) in undifferentiated and differentiated ESCs R1, EGCs-10, ECCs F9 and ECCs P19. (A, C, D, E, F) Uncropped Western blots for whole protein lysates from undifferentiated ESCs R1, EGCs-10, ECCs F9 and ECCs P19 - (1, 3, 5, 7 lanes) and after 5 day RA stimulation (2, 4, 6, 8 lanes, relatively). Prestained SDS-PAGE Broad Range Standard (161-0318, BioRad) was used. (B) Densitometry of bands intensity from (A). Plot shows relative optical density change of all bands in predicted area 85-100 KDa for undifferentiated and differentiating ESCs R1, EGCs EGC-10, ECCs F9 and ECCs P19. The relative protein expression levels were normalized to a-tubulin of corresponding samples from (F).

mouse Mage-d1 isoforms CRA_a	EDL29758.1; 592 aa
mouse Mage-d1 isoforms CRA_b	EDL29757.1, NP_062765.1; 775aa
mouse Mage-d2 isoforms X1	XP_006529062.1; 616 aa
mouse Mage-d2 isoform X2	XP_011246184.1; 594 aa
mouse Mage-e1	NP_444431; 918 aa
human MAGED1	XP_016885469.1; 778 aa
human MAGED2	NP_055414.2; 606 aa

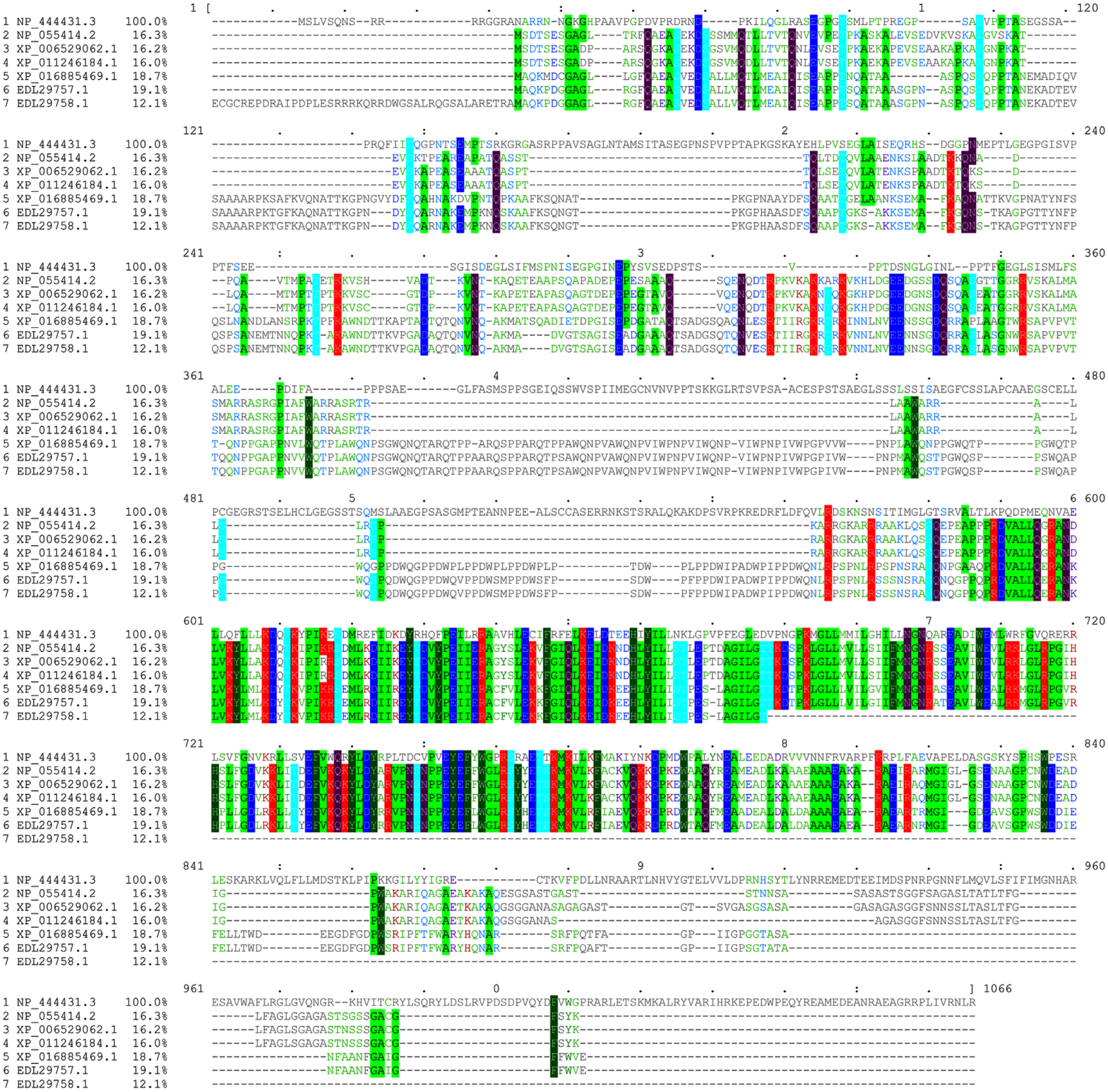

Supplementary Figure 3: Alignment of protein sequences of mouse Mage-d1, Mage-d2, Mage-e1 and human MAGED1 and MAGED1 using Clustal Omega Multiple Alignment Tool of the European Bioinformatics Institute (EMBL-EBI) (https://www.ebi.ac.uk/). All protein sequences analyzed contain several homologous regions-epitopes, which can be recognized by the antibodies against MAGED2/MADED (NBP2-24694, Novus Biologicals) and detected in western blots.

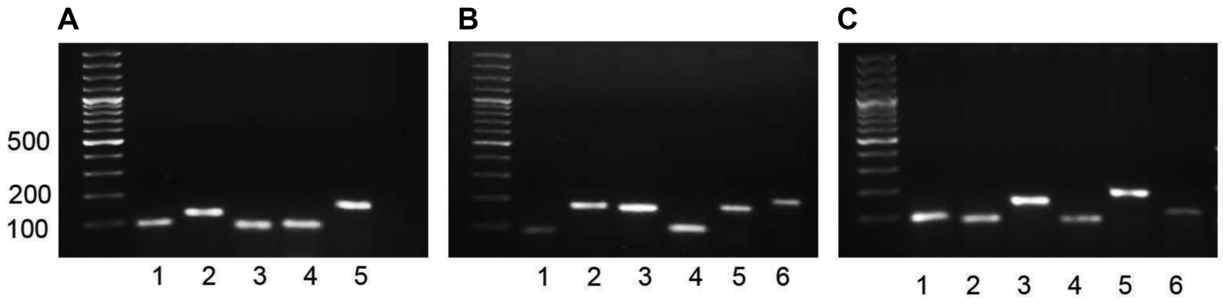

Supplementary Figure 4: Expression of the *Mage* family genes in adult mouse testes. PCR amplification of cDNA equivalent to 50 ng of total RNA from adult mouse testes was carried out in PCR mix containing 70 mM TpNc-HCI buffer, pH 8.6/25°C with 16.6 mM $(NH_4)_2SO_4$, 2.5 mM $MgCl_2$, 1.25 U Colored Taq DNA polymerase, 0.1 mM cresol red, (E0211, Silex, Russia), 0.1 mM dNTPs (Fermentas), and 0.2 microM of each primer were used at each reaction. PCR analysis of gene expression was carried out on an Eppendorf master cycler (Eppendorf, Germany) according to following program: preliminary denaturation at 94 degrees C for 5 min, 30 cycles, each PCR cycle consisted of 20 sec denaturing (94 degrees C), 20 sec annealing of primers (60 degress C), and 30 sec elongation (72 degress C) and final extension at 72°C for 10 min. All assays were conducted under identical conditions. The PCR products were then separayed on a 1.5% agarose gel and tris-borate buffer (0.5, pH 8.3), stained with ethidium bromide and registered using transilluminator (BioRed, USA) Designations: (**A**) 1 - Mage-a2, 2 - Mage-a4, 3 - Mage-a6, 4 - Mage-a8, 5 - Mage-a10; (**B**) 1 - Mage-b1, 2 - Mage-b3, 3 - Mage-b4, 4 - Mage-b5, 5 - Mage-b16, 6 - Mage-b18; (**C**) 1 - Mage-d1, 2 - Mage-d2, 3 - Mage-e1, 4 - Mage-e2, 5 - Mage-h1, 6 - Mage-12. The size of amplicons see in Supplementary Table 1.
